# Genome-Wide Identification and Evolutionary Analysis of *Gossypium* Tubby-Like Protein (TLP) Gene Family and Expression Analyses During Salt and Drought Stress

**DOI:** 10.3389/fpls.2021.667929

**Published:** 2021-07-21

**Authors:** Nasreen Bano, Shafquat Fakhrah, Chandra Sekhar Mohanty, Sumit Kumar Bag

**Affiliations:** ^1^Council of Scientific & Industrial Research-National Botanical Research Institute (CSIR-NBRI), Lucknow, India; ^2^Academy of Scientific and Innovative Research (AcSIR), Ghaziabad, India

**Keywords:** genome-wide analysis, transcription factor, expression, phylogenetic analysis, salt and drought stress responses, network

## Abstract

Tubby-like proteins (TLPs) possess a highly conserved closed β barrel tubby domain at C-terminal and N-terminal F-box. The role of *TLP* gene family members has been widely discussed in numerous organisms; however, the detailed genome-wide study of this gene family in *Gossypium* species has not been reported till date. Here, we systematically identified 105 *TLP* gene family members in cotton (*Gossypium arboreum, Gossypium raimondii, Gossypium hirsutum*, and *Gossypium barbadense*) genomes and classified them into eight phylogenetic groups. Cotton *TLP12* gene family members clustered into two groups, 4 and 8. They experienced higher evolutionary pressure in comparison to others, indicating the faster evolution in both diploid as well as in tetraploid cotton. Cotton *TLP* gene family members expanded mainly due to segmental duplication, while only one pair of tandem duplication was found in cotton *TLPs* paralogous gene pairs. Subsequent qRT-PCR validation of seven putative key candidate genes of *GhTLPs* indicated that *GhTLP11A* and *GhTLP12A.1* genes were highly sensitive to salt and drought stress. The co-expression network, pathways, and *cis*-regulatory elements of *GhTLP11A* and *GhTLP12A.1* genes confirmed their functional importance in salt and drought stress responses. This study proposes the significance of *GhTLP11A* and *GhTLP12A.1* genes in exerting control over salt and drought stress responses in *G. hirsutum* and also provides a reference for future research, elaborating the biological roles of *G. hirsutum TLPs* in both stress responses.

## Introduction

Tubby-like proteins are a family of bipartite transcription factors that were first studied in animals (Boggon et al., 1999; Santagata et al., 2001; Carroll et al., 2004) but have subsequently been identified from single-celled to multicellular organisms (Liu, 2008). Tubby-like proteins (TLPs) are characterized by the presence of the conserved C-terminal tubby domain, comprising of 12 anti-parallel closed β-barrel strands with a central hydrophobic α-helix (Boggon et al., 1999). In animals, TLPs are present in fewer numbers (ranging up to five) but have been ascribed to a wide range of cellular functions, including involvement in neuronal functions and development (Kleyn et al., 1996). In contrast to animals, the *TLP* family in plants is much larger with more than 10 members. Moreover, unlike animal TLPs, which possess a variable N-terminal region, plant TLP proteins possess a conserved N-terminal F-box domain besides the C-terminal tubby domain (Noben-Trauth et al., 1996; Gagne et al., 2002; Lai et al., 2004; Xu et al., 2016). F-box-comprising proteins are involved in the ubiquitin-mediated degradation of proteins (Kile et al., 2002), suggesting a role for TLPs in such processes.

In plants, tubby-like proteins have been studied in some dicot and monocots, such as *Arabidopsis thaliana*, poplar, rice (Yang Z. et al., 2008), apple (Xu et al., 2016), and maize (Chen et al., 2016). In *A. thaliana* (At), 11 *TLP* genes have been identified while 14, 15, and 10 genes have been identified in rice, maize, and apple (Yang Z. et al., 2008; Chen et al., 2016; Xu et al., 2016). Studies of the *TLP* families in these plants reveal expression in different tissues and in response to different hormone treatments or under abiotic stress conditions (Lai et al., 2004; Liu, 2008; Xu et al., 2016). In *Arabidopsis, AtTLP3*, and *AtTLP9* were found to function redundantly in response to abscisic acid (ABA) and osmotic stress treatments (Lai et al., 2004), while *AtTLP9* was also demonstrated to respond in drought and salt stress (Lai et al., 2004; Bao et al., 2014). In apple, several *TLP* genes were upregulated in response to abiotic stress treatments, suggesting an important role for *TLP* genes in stress responses (Xu et al., 2016). In *Cicer arietinum, CaTLP1* was expressed in response to dehydration stress, and its expression in tobacco led to enhanced tolerance to drought, oxidative, and salt stress (Wardhan et al., 2012). Collectively, these studies suggest that TLPs might have a significant function in the stress response of diverse plant species (Wang et al., 2018). However, the role of plants *TLPs* and their mode of action remain largely unknown (Zhang et al., 2020).

Cotton (*Gossypium* spp.) is the most important natural fiber producing crop worldwide (Yang et al., 2020a). A lot of diversity exists in the *Gossypium* genus that includes six tetraploid species (2n = 52) and 45 diploids (2n = 26) (Hawkins et al., 2006; Grover et al., 2015). Interspecific hybridization events among the *G. herbaceum* (A1) or *G. arboreum* (A2) (A-genome ancestral African species) and *G. raimondii* or *G. gossypioides* (D6) (D-genome native species) have resulted in allotetraploid *G. hirsutum* (upland cotton) and *G. barbadense* (Senchina et al., 2003; Zhu and Li, 2013), which are possibly the oldest main allopolyploid crops (Paterson et al., 2012; Chalhoub et al., 2014; Marcussen et al., 2014). Diversity within the *Gossypium* species provides a perfect model for examining the evolution and polyploid domestication (Yang et al., 2020a) and has been facilitated further with the completion of the whole genome sequences of *G. raimondii, G. arboreum, G. barbadense*, and *G. hirsutum* in the last few years (Wang K. B. et al., 2012; Li et al., 2014; Liu X. et al., 2015; Zhang et al., 2015). The large evolutionary diversity in cotton allows it to adapt to several different types of regions with differing environmental conditions, although the molecular basis of this adaptation is not yet well-understood.

We are interested in the evolution of the cotton *TLP* gene family and their possible roles in abiotic stress responses. So far, only a single member of the family *GhTULP34* has been characterized (Li et al., 2020). In this study, we have carried out comprehensive genomic exploration of TLP protein family in *G. raimondii, G. arboreum, G. barbadense*, and *G. hirsutum* and studied the expression profiles of *G. hirsutum TLPs* (*GhTLPs*) in salt and drought stress responses. The aim of this study was to provide a comprehensive understanding of cotton *TLP* genes for future breeding programs for the improvement of plant quality, production, and response to abiotic stresses.

## Materials and Methods

### Identification of the *TLP* Gene Family in *Gossypium* Species

The protein, cDNA, gene annotation, and genome files (gff) of *G. arboreum* (BGI_A2 v1)*, G. raimondii* (JGI v2), *G. barbadense* (HAU v2), and *G. hirsutum* (HAU v1) were retrieved from the CottonGen resources (Yu et al., 2014) while the protein sequence of *A. thaliana* was procured from the TAIR database (Lamesch et al., 2012). The Hidden Markov Model (HMM) profiles of the TLP domain (PF01167) were taken from the Pfam database (El-Gebali et al., 2019). The four cotton genomes were employed as queries, and the Pfam database was used as a reference to identify the TLP protein in the cotton dataset, using the HMMER search program (http://hmmer.wustl.edu/; Eddy, 2011). The identified *TLP* genes were further confirmed by BLASTP search and NCBI Batch-CD search (Lu et al., 2020).

### Physiochemical Properties and Characterization of Cotton *TLP* Genes

The physiochemical properties, such as charge, molecular weight (Da), grand average of hydropathy (GRAVY), instability index, isoelectric points (pI) of *G. arboreum* (Ga), *G. raimondii* (Gr), *G. barbadense* (Gb), and *G. hirsutum* (Gh) TLPs, were determined through the ProtParam tool in the ExPASy web server (Gasteiger et al., 2003). The subcellular localization of cotton TLP proteins was predicted by using the software CELLO v.2.5 (Yu et al., 2006).

### Analysis of the Encoded Protein Motif, Gene Structure, and miRNA Target Sites of Cotton TLP Proteins

The conserved protein motifs of cotton *TLP* genes were identified through the MEME (Multiple Em for Motif Elicitation) version 5.0.1 (Bailey et al., 2009) by employing the full-length proteins encoded by *TLP* genes in cotton. The exon-intron structure analysis was carried out with Gene Structure Display Server 2.0 (Guo et al., 2007), using the genomic and coding sequences of the identified cotton *TLPs*. To decipher the miRNA target sites in the cotton TLP transcripts, the complete sequences of all known and reported miRNAs of the four cotton species were fetched from the miRBase database (http://www.mirbase.org/; Kozomara et al., 2019). miRNA target site prediction analysis was performed through a plant small RNA target analysis server (psRNATarget 2017 release) (Dai et al., 2018), using the 376 cotton miRNA sequences.

### Multiple Sequence Alignments (MSA) and Phylogenetic Analysis

To identify conserved regions of predicted cotton TLP proteins, multiple sequence alignments (MSA) were performed with the ClustalX2.1 program (Larkin et al., 2007), using default criteria. Phylogenetic tree construction was carried out through MEGA7 software (Kumar et al., 2016), with the maximum likelihood (ML) method, using the 1,000 bootstrap and the Jones-Taylor-Thornton (JTT) model. Visualization of the tree was carried out through Interactive Tree Of Life (iTOL; Letunic and Bork, 2019).

### Gene Duplication Event, Chromosomal Distribution, and Synteny Analysis

To know the evolutionary mechanism of *TLP* gene in *Gossypium*, the paralogous *TLP* genes were identified in *G. arboreum, G. raimondii, G. barbadense*, and *G. hirsutum*, using a reciprocal blast with e-value 10^−5^. Paralogous genes are described as similarity of the aligned regions >70% and shared aligned region covering >70% of the gene length (Yang S. et al., 2008). The *Ka/Ks* ratio of orthologous and paralogous sequences was identified through the PAL2NAL program (Suyama et al., 2006), which was further used to compute the approximate date of duplication and divergence events with the formula T = Ks/2λ, assuming the clocklike rate (λ) of 1.5 synonymous substitutions per 10^−8^ years for cotton (Blanc and Wolfe, 2004; Tang et al., 2016). Moreover, the *Ka/Ks* ratio was also employed to show the selection pressure for the duplicated *TLP* genes. A *Ka/Ks* = 1, >1, and <1 demonstrate neutral, positive, and negative (purifying selection) evolution, respectively. Orthologous of *TLP* genes of cotton with *A. thaliana* and *Theobroma cacao* were identified *via* a reciprocal blast with an e-value 10^−5^. As per the result of the reciprocal blast, duplication events, and syntenic blocks of cotton *TLP* genes were detected through McScanX, and visualization of orthologous *TLP* genes between cotton (*G. raimondii, G. arboreum, G. barbadense*, and *G. hirsutum*) and two other species (*A. thaliana* and *T. cacao*) was performed through CIRCOS (Krzywinski et al., 2009; Wang Y. P. et al., 2012). The chromosomal location of all cotton *TLP* genes was found through the BLASTN search program on TLPs CDS sequences against the CottonGen (https://www.cottongen.org/) database. Total cotton *TLP* genes were mapped on the chromosome through Mapinspect software (http://mapinspect.software.informer.com/).

### Expression Profile of Cotton *TLP* Gene Family Members Under Salt and Drought Stress Conditions

To gain insight into the expression profile of cotton *TLP* gene family members under salt and drought stress conditions, the Illumina RNA-Seq data of *G. hirsutum* (accession number: PRJNA532694) were retrieved from the NCBI database. The poor-quality reads were filtered by Fastx-toolkit (Schmieder and Edwards, 2011) and mapped to the *G. hirsutum* genome, using the TopHat2 (Kim et al., 2013). The estimation of transcript abundance was carried out with fragments per kilobase per million (FPKM) through Cufflinks software (Trapnell et al., 2012). The hierarchical clustered heatmap generation and visualization were done in the R program, using the pheatmap package.

### RNA Isolation, cDNA Preparation, and Quantitative Real-Time PCR (qRT-PCR) Validation

The selected putative *TLP* genes were validated in 2-month-old drought and salt-stressed plants of *G. hirsutum*, grown in the field under normal photoperiodic conditions. Plants were grown in triplicate, and a single treatment of 300-mM NaCl was used to stimulate salt stress (Wei et al., 2017) and 20% PEG8000 solution to decrease the osmotic potential of the root, inducing drought stress (Shafiq et al., 2015). The non-treated plants were taken as control. Leaf tissues were collected at 6, 12, 24, 48, and 72 h after treatment, RNA isolated as per the protocol (Sigma USA), followed by cDNA (1 μg/μl) synthesis with the verso cDNA synthesis kit (Thermo scientific) as per the provided protocol. Expression of seven genes was checked by qRT-PCR fluorescent quantitative detection system (HiMedia Insta Q 48 M4), using fast the SYBER^TM^ green master mix (Applied Biosystem) with primers designed with the help of primer express 3.0. Ubiquitin was taken as the internal control. The reaction conditions of qRT-PCR were 95°C for 10 min, followed by cycling for 40 cycles of denaturation at 95°C for 10 s, annealing at 56°C for 15 s and extension at 72°C for 30 s. Relative expression of the employed genes was calculated with mean ± SD of biological triplicate samples by the 2–^ΔΔ^Ct method (Livak and Schmittgen, 2001).

### Co-expression Network and Metabolic Pathway Analysis of Negatively and Positively Co-expressed Genes With *GhTLP11A* and *GhTLP12A.1*

The co-expression network of the *GhTLP11A* and *GhTLP12A.1* genes in salt and drought stress conditions was constituted by the FPKM values with the “expression correlation networks” module in Cytoscape version 3.8.0 (Smoot et al., 2011). The module calculated positive Pearson correlations (*r* ≥ 0.95) and negative correlations (*r* ≤ −0.95), with interacting members of the network. Network visualization and co-expression of genes were shown in Cytoscape by applying the force-directed layout. The important metabolic pathways and functional categories of positively and negatively co-expressed genes (PCoEGs and NCoEGs) with *GhTLP11A* and *GhTLP12A.1* were estimated, using the MapMan software 3.5.1 version (Thimm et al., 2004). The average statistical test accompanied by the Benjamini Hochberg (multiple correction tests) was employed to know the functional categories.

### Identification of *cis*-Regulatory Elements of *GhTLP* Genes and Homology Modeling of the Highly Expressed *GhTLPs*

The 2-Kb sequences upstream of *GhTLP* genes was analyzed for *cis*-regulatory elements by using the PlantCARE database (Lescot et al., 2002) by the “Signal Scan Search” program. The three-dimensional structure of GhTLPs was obtained through homology modeling, using the Phyre2 (Kelley et al., 2015) server. Structure visualization of GhTLPs was carried out with Chimera 1.11.1 version (Pettersen et al., 2004).

### Statistical Analysis

The statistical analysis of qRT-PCR was carried out, using GraphPad Prism version 8.4.3 software, with two-tailed Student's *T*-tests in triplicate sample repeats.

## Results

### Genome-Wide Identification of *TLP* Genes in *Gossypium* Species

The protein sequences of *G. arboreum, G. raimondii, G. hirsutum*, and *G. barbadense* were utilized to identify cotton *TLP* genes. The identified *TLP* genes were confirmed through conserved domain searches. A total of 105 *TLPs*, i.e., 19 *GaTLPs* (*G. arboreum*), 18 *GrTLPs* (*G. raimondii*), 33 *GhTLPs* (*G. hirsutum*), and 35 *GbTLPs* (*G. barabadense*) were determined (Table 1). The length of cotton TLP proteins varied from 68 to 425 amino acid residues (aa) in *G. arboreum*, 320 to 519 aa in *G. raimondii*, 206 to 514 aa in *G. hirsutum*, and 206 to 494 aa in *G. barabadense*. The theoretical isoelectric point (pI) ranged from 5.1 to 9.7, 9 to 9.3, 8.3 to 9.8, and 7.6 to 9.8**;** the molecular weight ranged approximately from 8 to 48 kDa, 36 to 58 kDa, 23 to 58 kDa, and 23 to 55 kDa, and the number of introns ranged from 0 to 7, 4 to 8, 2 to 8, and 0 to 8 in *G. arboreum, G. raimondii, G. barabadense*, and *G. hirsutum*, respectively. Most of the identified cotton TLP proteins were predicted to be nuclear localized, and others were likely localized in extracellular space, mitochondrion, and on plasma membrane. For the annotation of 105 identified cotton *TLP* genes, the *A. thaliana* nomenclature system was pursued with numbers representing the highest sequence similarity with the corresponding *AtTLP* orthologous. Accordingly, the 19 *GaTLPs* were named as *GaTLP2-GaTLP12* (*GaTLP2.1, 2.2, 2.3, 2.4, 5.1, 5.2, 5.3, 6.1, 6.2, 6.3, 7.1, 7.2, 8, 11, 12.1, 12.2, 12.3, 12.4*, and *12.5*), *GrTLPs* were classified as *GrTLP2-GrTLP12* (*GrTLP2.1, 2.2, 2.3, 5.1, 5.2, 5.3, 6.1, 6.2, 6.3, 7.1, 7.2, 7.3, 8, 11, 12.1, 12.2, 12.3*, and *12.4*). Similarly, *GhTLPs* and *GbTLPs* were named *GhTLP2-GhTLP12A/D* and *GbTLP2-GbTLP12A/D* (A: At subgenome and D: Dt subgenome). The reciprocal blast demonstrated that cotton *TLP* genes showed greater homology with *AtTLP2, AtTLP5, AtTLP6, AtTLP7*, and *AtTLP8* as compared with *AtTLP1, AtTLP3, AtTLP4, AtTLP9, AtTLP10*, and *AtTLP11*, respectively (Supplementary Table 1).

**Table 1:** Characteristics of *TLP* genes in cotton.

**Gene name**	**Gene ids**	**Chromosome location**	**Length**	**Molecular weight (Da)**	**pI**	**No. of intron**	**Subcellular localization**	**Negatively charged residues (Asp + Glu)**	**Positively charged residues (Arg + Lys)**	**Instability index**	**Stability**	**Aliphatic index**	**Grand average of hydropathicity (GRAVY)**
GaTLP5.1	Cotton_A_33944	CA_chr10:6955456:6958125:–	424	47579.4	9.47	3	Nuclear	41	59	61.21	Unstable	76.13	−0.393
GaTLP11	Cotton_A_08277	CA_chr4:25409214:25412052:–	405	45396.09	9.38	4	Nuclear	34	52	57.93	Unstable	75.06	−0.32
GaTLP5.2	Cotton_A_33887	CA_chr2:80696652:80698513:+	421	47075.12	9.59	5	Nuclear	36	57	54.47	Unstable	79.64	−0.284
GaTLP5.3	Cotton_A_00581	CA_chr5:8963634:8965537:+	425	47770.92	9.66	5	Nuclear	38	60	60.61	Unstable	77.53	−0.327
GaTLP2.1	Cotton_A_02013	CA_chr6:29518659:29520756:+	414	46389.2	9.15	4	Nuclear	41	53	61.74	Unstable	78.43	−0.35
GaTLP12.2	Cotton_A_34069	CA_chr1:125845208:125847603:+	376	41970.32	9.44	4	Nuclear	28	45	55.82	Unstable	80.96	−0.276
GaTLP12.3	Cotton_A_36302	CA_chr11:88651667:88653976:+	393	43586.25	9.55	5	Nuclear	30	51	59.31	Unstable	79.41	−0.223
GaTLP6.2	Cotton_A_06847	CA_chr7:115616747:115618648:–	407	45540.23	9.56	5	Nuclear	35	55	62.21	Unstable	76.88	−0.335
GaTLP6.1	Cotton_A_13768	CA_chr8:15732526:15733950:–	393	43846.49	9.74	4	Nuclear	32	56	54.37	Unstable	74.2	−0.38
GaTLP12.1	Cotton_A_29560	CA_chr5:44377749:44379416:+	343	38292.16	9.48	4	Nuclear	26	42	49.07	Unstable	82.48	−0.237
GaTLP7.1	Cotton_A_20151	CA_chr3:24210746:24214201:+	366	40348.79	9.33	4	Nuclear	31	46	55.84	Unstable	70.66	−0.37
GaTLP7.2	Cotton_A_29419	CA_chr7:92995264:92998667:–	389	43679.8	9.16	3	Nuclear	40	53	60.89	Unstable	66.97	−0.49
GaTLP2.2	Cotton_A_17032	CA_chr9:56110460:56112674:–	403	45105.88	8.81	4	Nuclear	41	49	62.52	Unstable	78.41	−0.308
GaTLP6.3	Cotton_A_18767	CA_chr10:46424178:46425914:+	423	46938.87	9.66	5	Nuclear	36	61	59.72	Unstable	74.04	−0.418
GaTLP2.3	Cotton_A_14780	CA_chr6:87318145:87320189:–	403	45344.18	9.6	8	Nuclear	39	58	68.88	Unstable	84.69	−0.375
GaTLP8	Cotton_A_17106	CA_chr10:88944897:88947133:+	417	46630.12	9.3	4	Nuclear	39	56	41.81	Unstable	71.58	−0.56
GaTLP2.4	Cotton_A_01603	CA_ch1:135480257:135480915:+	197	22296.62	8.56	4	Plasma Membrane	15	18	58.85	Unstable	77.26	−0.042
GaTLP12.4	Cotton_A_22539	CA_chr10:13654011:13655817:+	273	30954.9	9.71	5	Nuclear	18	36	42.07	Unstable	85.68	−0.27
GaTLP12.5	Cotton_A_22538	CA_chr10:13655845:13656051:+	68	7867.1	5.1	5	Extracellular	5	6	36.17	Stable	89.09	0.213
GrTLP7.3	Gorai.002G054300.1	GR_chr02:4808034:4811930:+	389	43453.59	9.16	4	Nuclear	39	52	62.86	Unstable	69.23	−0.444
GrTLP5.2	Gorai.002G207100.1	GR_chr02:55351835:55355046:–	421	47047.05	9.64	0	Nuclear	36	57	55.11	Unstable	79.41	−0.295
GrTLP12.4	Gorai.004G085800.1	GR_chr04:10773031:10775214:+	397	44814.74	9.43	5	Nuclear	32	52	52.32	Unstable	80.76	−0.265
GrTLP7.2	Gorai.004G271800.1	GR_chr04:60665497:60669265:+	384	42453.18	9.3	4	Nuclear	34	49	57.19	Unstable	68.62	−0.437
GrTLP12.1	Gorai.005G057200.1	GR_chr05:5803516:5805608:+	384	42875.25	9.27	3	Nuclear	34	48	48.73	Unstable	82.55	−0.305
GrTLP5.3	Gorai.005G259600.1	GR_chr05:63517604:63520935:+	425	47759.91	9.66	5	Nuclear	38	60	62.8	Unstable	75.69	−0.348
GrTLP12.3	Gorai.006G075400.1	GR_chr06:29765762:29768708:+	413	46131.89	9.63	3	Nuclear	33	54	61.19	Unstable	76.95	−0.306
GrTLP2.2	Gorai.007G050300.1	GR_chr07:3545878:3549332:+	414	46419.21	9.19	4	Nuclear	41	53	62.06	Unstable	78.19	−0.352
GrTLP11	Gorai.007G131500.1	GR_chr07:10627794:10632340:–	405	45454.24	9.38	5	Nuclear	34	52	57.21	Unstable	75.09	−0.313
GrTLP2.3	Gorai.008G067400.1	GR_chr08:10983416:10986428:+	417	46564.64	9.18	8	Nuclear	41	54	63.56	Unstable	83.26	−0.294
GrTLP8	Gorai.009G121900.1	GR_chr09:9059982:9062702:+	320	36316.03	9.63	5	Mitochondrial	26	46	37.99	Unstable	76.19	−0.452
GrTLP6.1	Gorai.009G201100.1	GR_chr09:15567676:15569918:+	409	45707.86	9.8	4	Nuclear	32	59	52.16	Unstable	77.02	−0.336
GrTLP5.1	Gorai.009G254100.1	GR_chr09:20878526:20882901:+	400	44857.66	9.75	5	Nuclear	33	57	62.18	Unstable	78.75	−0.352
GrTLP6.3	Gorai.009G272900.1	GR_chr09:22824350:22826996:+	519	57823.54	9.69	4	Nuclear	44	72	61.61	Unstable	78.94	−0.287
GrTLP7.1	Gorai.009G367200.1	GR_ch09:49203689:49207350:+	395	43456.41	9.2	5	Nuclear	39	53	63.6	Unstable	68.68	−0.414
GrTLP6.2	Gorai.010G009400.1	GR_chr10:705149:707577:+	413	46131.89	9.63	4	Nuclear	33	54	61.19	Unstable	76.95	−0.306
GrTLP2.1	Gorai.011G101400.1	GR_chr11:11429229:11432319:+	409	45774.66	9	4	Nuclear	41	51	60.14	Unstable	78.92	−0.325
GrTLP12.2	Gorai.011G185600.1	GR_chr11:44201423:44204423:+	376	41963.24	932	3	Nuclear	29	44	56.39	Unstable	80.19	−0.294
GhTLP7A.2	Ghir_A01G004680.1	GhA:chr01:6048661:6051724:+	389	43580.66	9.16	4	Nuclear	40	53	62.52	Unstable	66.97	−0.49
GhTLP5A.1	Ghir_A01G016830.1	GhA:chr01:107578137:107581303:–	420	46917.95	9.59	4	Nuclear	35	56	54.56	Unstable	79.83	−0.274
GhTLP5A.2	Ghir_A03G022780.1	GhA:chr03:112389242:112392899:+	425	47784.9	9.66	5	Nuclear	38	60	60.77	Unstable	76.61	−0.34
GhTLP8A	Ghir_A05G012060.1	GhA:chr05:11062311:11064976:+	417	46640.15	9.3	3	Nuclear	39	56	40.21	Unstable	71.58	−0.546
GhTLP6A.1	Ghir_A05G019690.1	GhA:chr05:18821376:18823547:+	406	45493.57	9.83	3	Nuclear	32	59	52.23	Unstable	74.21	−0.373
GhTLP6A.3	Ghir_A05G026280.1	GhA:chr05:27453966:27457122:+	418	46601.68	9.73	4	Nuclear	35	62	56.1	Unstable	74	−0.417
GhTLP6A.2	Ghir_A06G000860.1	GhA:chr06:869775:871665:+	407	45473.13	9.51	3	Nuclear	35	54	62.9	Unstable	77.13	−0.332
GhTLP7A.1	Ghir_A08G024660.1	GhA:chr08:120995211:120998711:+	381	42239.9	9.17	3	Nuclear	36	49	56.33	Unstable	67.87	−0.449
GhTLP12A.2	Ghir_A09G006930.1	GhA:chr09:53138712:53141783:+	393	43627.33	9.5	3	Nuclear	30	50	57.78	Unstable	77.68	−0.217
GhTLP2A.1	Ghir_A10G009400.1	GhA:chr10:18951476:18953688:–	383	43070.52	8.61	3	Nuclear	39	44	63.11	Unstable	81.23	−0.283
GhTLP12A.1	Ghir_A10G010240.1	GhA:chr10:21759983:21762837:–	376	42012.42	9.38	4	Nuclear	28	45	55.28	Unstable	80.96	−0.262
GhTLP2A.2	Ghir_A11G004920.1	GhA:chr11:4252195:4256299:+	414	46399.22	9.19	3	Nuclear	41	53	63.16	Unstable	79.37	−0.346
GhTLP11A	Ghir_A11G012590.1	GhA:chr11:12921875:12926492:–	311	34589.58	9.27	2	Nuclear	24	37	54.41	Unstable	76.5	−0.316
GhTLP2A.3	Ghir_A12G006600.1	GhA:chr12:15242985:15244849:+	393	44061.57	9.08	8	Nuclear	39	50	66.98	Unstable	81.37	−0.317
GhTLP7D.3	Ghir_D01G004710.1	GhD:chr01:5448473:5453121:+	416	46531.15	9.26	4	Nuclear	43	58	57.73	Unstable	68.73	−0.454
GhTLP5D.2	Ghir_D01G018390.1	GhD:chr01:55366719:55369873:–	421	47017.02	9.64	4	Nuclear	36	57	55.57	Unstable	79.64	−0.289
GhTLP12D.1	Ghir_D02G005060.1	GhD:chr02:6472304:6473565:+	277	30665.32	9.62	4	Nuclear	19	33	45.77	Unstable	84.19	−0.17
GhTLP5D.3	Ghir_D02G024220.1	GhD:chr02:69191552:69195172:+	425	47759.91	9.66	5	Nuclear	38	60	62.8	Unstable	75.69	−0.348
GhTLP2D.4	Ghir_D03G001340.1	GhD:chr03:950448:951272:+	206	22753.18	8.35	3	Plasma Membrane	18	20	57.67	Unstable	88.16	0.034
GhTLP8D	Ghir_D05G011810.1	GhD:chr05:10003116:10005943:+	417	46623.22	9.39	4	Nuclear	36	55	41.38	Unstable	72.06	−0.514
GhTLP6D.1	Ghir_D05G019700.1	GhD:chr05:17145578:17150416:+	414	46373.8	9.84	4	Extracellular	30	58	47.79	Unstable	81.74	−0.246
GhTLP5D.1	Ghir_D05G024720.1	GhD:chr05:22834369:22839003:+	424	47545.38	9.47	5	Nuclear	41	59	61.21	Unstable	77.05	−0.391
GhTLP6D.3	Ghir_D05G026320.1	GhD:chr05:24926304:24929436:+	514	57545.28	9.73	4	Nuclear	44	73	59.56	Unstable	77.06	−0.301
GhTLP7D.1	Ghir_D05G034870.1	GhD:chr05:53632702:53635785:+	381	41802.58	9.33	5	Nuclear	35	51	68.57	Unstable	70.21	−0.393
GhTLP6D.2	Ghir_D06G000730.1	GhD:chr06:707815:710329:+	406	45352	9.59	4	Nuclear	34	54	62.54	Unstable	76.82	−0.325
GhTLP12D.4	Ghir_D08G007820.1	GhD:chr08:11289331:11291206:+	304	34460.84	9.68	5	Nuclear	22	42	51.33	Unstable	78.19	−0.337
GhTLP7D.2	Ghir_D08G025550.1	GhD:chr08:67215180:67218700:+	383	42389.13	9.39	4	Nuclear	34	50	57.46	Unstable	69.32	−0.449
GhTLP12D.3	Ghir_D09G006640.1	GhD:chr09:30300709:30303722:+	393	43548.16	9.6	4	Nuclear	29	51	57.01	Unstable	78.17	−0.231
GhTLP2D.1	Ghir_D10G009850.1	GhD:chr10:12012251:12016560:+	413	46283.13	8.73	4	Nuclear	45	53	58.13	Unstable	78.43	−0.327
GhTLP12D.2	Ghir_D10G017760.1	GhD:chr10:48294734:48297960:+	376	41991.29	9.32	4	Nuclear	29	44	56.57	Unstable	80.72	−0.289
GhTLP2D.2	Ghir_D11G004820.1	GhD:chr11:3929455:3933050:+	414	46393.13	9.19	4	Nuclear	41	53	63.59	Unstable	77.25	−0.363
GhTLP11D	Ghir_D11G012540.1	GhD:chr11:11519838:11525175:–	405	45398.17	9.37	4	Nuclear	34	52	55.54	Unstable	74.84	−0.312
GhTLP2D.3	Ghir_D12G006610.1	GhD:chr12:11431068:11434161:+	417	46744.74	9.28	8	Nuclear	41	55	63.43	Unstable	81.85	−0.333
GbTLP7A.3	Gbar_A01G004500.1	GbA:chr01:5828899:5832744:+	389	43607.69	9.16	4	Nuclear	40	53	62.02	Unstable	66.97	−0.497
GbTLP5A.2	Gbar_A01G017260.1	GbA:chr01:105676110:105677971:–	421	47105.21	9.59	5	Nuclear	36	57	54.47	Unstable	79.64	−0.278
GbTLP12A.1	Gbar_A02G004650.1	GbA:chr02:6142192:6146051:+	384	42685.15	9.37	3	Nuclear	32	48	49.99	Unstable	81.54	−0.239
GbTLP5A.3	Gbar_A03G022950.1	GbA:chr03:104669910:104676138:+	425	47726.82	9.61	4	Nuclear	38	59	60.89	Unstable	76.61	−0.337
GbTLP7A.1	Gbar_A04G003920.1	GbA:chr04:9544967:9548909:–	387	42650.7	9.26	3	Nuclear	37	52	66.36	Unstable	69.61	−0.406
GbTLP8A.2	Gbar_A05G011460.1	GbA:chr05:10538169:10540756:+	417	46624.15	9.31	5	Nuclear	39	56	40.21	Unstable	71.58	−0.536
GbTLP6A.1	Gbar_A05G019030.1	GbA:chr05:18030893:18033198:+	409	45746.85	9.83	4	Nuclear	32	59	53.05	Unstable	75.82	−0.354
GbTLP6A.3	Gbar_A05G025320.1	GbA:chr05:26213749:26217047:+	494	55077.28	9.56	4	Nuclear	42	66	59.29	Unstable	76.42	−0.29
GbTLP8A.1	Gbar_A05G043170.1	GbA:Scaffold3378:17019:19596:+	417	46623.22	9.39	6	Nuclear	36	55	41.38	Unstable	72.06	−0.514
GbTLP5A.1	Gbar_A05G043600.1	GbA:Scaffold91:25302:29797:+	398	44752.25	9.49	4	Nuclear	38	56	64.05	Unstable	75.98	−0.373
GbTLP6A.2	Gbar_A06G000760.1	GbA:chr06:761708:763929:+	414	46249.04	9.56	3	Nuclear	34	54	60.4	Unstable	77	−0.298
GbTLP7A.2	Gbar_A08G025570.1	GbA:chr08:118191404:118195120:+	384	42596.32	9.23	3	Nuclear	36	50	57.61	Unstable	68.36	−0.449
GbTLP12A.3	Gbar_A09G007080.1	GbA:chr09:50278877:50281768:+	393	43609.3	9.5	4	Nuclear	30	50	57.98	Unstable	78.68	−0.21
GbTLP2A.1	Gbar_A10G010240.1	GbA:chr10:18997847:19001328:–	409	45839.81	8.89	4	Nuclear	41	50	63.81	Unstable	79.17	−0.296
GbTLP12A.2	Gbar_A10G011070.1	GbA:chr10:21723033:21724859:–	287	31681.29	9.49	1	Nuclear	19	33	49.42	Unstable	71.78	−0.366
GbTLP2A.2	Gbar_A11G004480.1	GbA:chr11:3813200:3817322:+	414	46399.22	9.19	2	Nuclear	41	53	63.16	Unstable	79.37	−0.346
GbTLP11A	Gbar_A11G012270.1	GbA:chr11:12405505:12409866:–	405	45396.09	9.38	4	Nuclear	34	52	57.93	Unstable	75.06	−0.32
GbTLP2A.3	Gbar_A12G006580.1	GbA:chr12:15078028:15080997:+	406	45532.2	9.24	8	Nuclear	41	54	69.05	Unstable	81.18	−0.354
GbTLP7D.3	Gbar_D01G004700.1	GbD:chr01:5641258:5644642:+	389	43453.59	9.16	5	Nuclear	39	52	62.86	Unstable	69.23	−0.444
GbTLP5D.2	Gbar_D01G018480.1	GbD:chr01:55332523:55334385:–	421	47065.07	9.64	4	Nuclear	36	57	55.59	Unstable	78.95	−0.292
GbTLP12D.1	Gbar_D02G005180.1	GbD:chr02:6638191:6639988:+	385	43023.52	9.23	4	Nuclear	35	49	50.31	Unstable	84.36	−0.267
GbTLP5D.3	Gbar_D02G024820.1	GbD:chr02:67069705:67071604:+	425	47836.02	9.58	5	Nuclear	38	59	61.97	Unstable	75.69	−0.333
GbTLP2D.4	Gbar_D03G001400.1	GbD:chr03:914036:914860:+	206	22731.12	7.62	4	Plasma Membrane	19	20	57.95	Unstable	88.16	0.033
GbTLP6D.1	Gbar_D05G019720.1	GbD:chr05:17031049:17033198:+	409	45823.04	9.88	3	Nuclear	32	61	51.17	Unstable	77.73	−0.341
GbTLP5D.1	Gbar_D05G024660.1	GbD:chr05:22759705:22764214:+	424	47545.38	9.47	5	Nuclear	41	59	61.21	Unstable	77.05	−0.391
GbTLP6D.3	Gbar_D05G026200.1	GbD:chr05:24657207:24660514:+	420	46692.68	9.75	7	Nuclear	35	61	59.71	Unstable	74.55	−0.393
GbTLP7D.1	Gbar_D05G034930.1	GbD:chr05:53368297:53371951:+	395	43414.33	9.2	5	Nuclear	39	53	65.22	Unstable	68.2	−0.42
GbTLP6D.2	Gbar_D06G000830.1	GbD:chr06:735015:737233:+	413	46105.85	9.63	3	Nuclear	33	54	59.66	Unstable	77.19	−0.298
GbTLP7D.2	Gbar_D08G026180.1	GbD:chr08:64380995:64384741:+	384	42454.17	9.24	4	Nuclear	35	49	56.8	Unstable	68.62	−0.437
GbTLP12D.3	Gbar_D09G006810.1	GbD:chr09:28809892:28812761:+	393	43591.22	9.59	4	Nuclear	29	51	56.39	Unstable	77.91	−0.242
GbTLP2D.1	Gbar_D10G009570.1	GbD:chr10:11447225:11450694:+	409	45693.54	8.92	5	Nuclear	41	50	61.68	Unstable	79.41	−0.309
GbTLP12D.2	Gbar_D10G017400.1	GbD:chr10:45988943:45991475:+	376	41977.27	9.32	3	Nuclear	29	44	56.57	Unstable	80.45	−0.286
GbTLP2D.2	Gbar_D11G004820.1	GbD:chr11:3795097:3797188:+	414	46427.15	9.19	4	Nuclear	41	53	63.59	Unstable	76.3	−0.366
GbTLP11D	Gbar_D11G012860.1	GbD:chr11:11271540:11276030:–	405	45398.17	9.37	5	Nuclear	34	52	55.54	Unstable	74.84	−0.312
GbTLP2D.3	Gbar_D12G006630.1	GbD:chr12:11251940:11254140:+	417	46744.74	9.28	3	Nuclear	41	55	63.43	Unstable	81.85	−0.333

### Domain Structure Analysis of TLP Protein Family Members in Cotton

All the cotton TLP proteins were predicted to contain the tubby domain at the C-terminal end. With the exception of some (GaTLP2.4, GaTLP7.1, GaTLP7.2, GaTLP8, GaTLP12.1, GaTLP12.4, GaTLP12.5, GrTLP7.1, GrTLP7.2, GrTLP7.3, GrTLP8, GhTLP8A, GhTLP2D.1, GhTLP2D.4, GhTLP2A.1, GhTLP7A.1, GhTLP7A.2, GhTLP7D.1, GhTLP7D.2, GhTLP11A, GhTLP12A.1, GhTLP12D.1, GhTLP12D.2, GhTLP12D.4, GbTLP2D.4, GbTLP7A.1, GbTLP7A.2, GbTLP7A.3, GbTLP7D.1, GbTLP7D.2, GbTLP7D.3, GbTLP8A.1, GbTLP8A.2, GhTLP8D, GbTLP12A.1, GbTLP12A.2, and GbTLP12.1), the majority of the proteins encoded by the cotton *TLP* genes also possessed an F-box domain (Supplementary Figure 1). In *A. thaliana*, the TLP8 also has the tubby domain at the C-terminal side but lacked F-box at the N-terminal side (Lai et al., 2004). This finding showed that cotton TLP proteins comprised the same domain arrangements as reported earlier (Lai et al., 2004).

### Multiple Sequence Alignment (MSA) and Evolutionary Analysis

**Figure 1:**
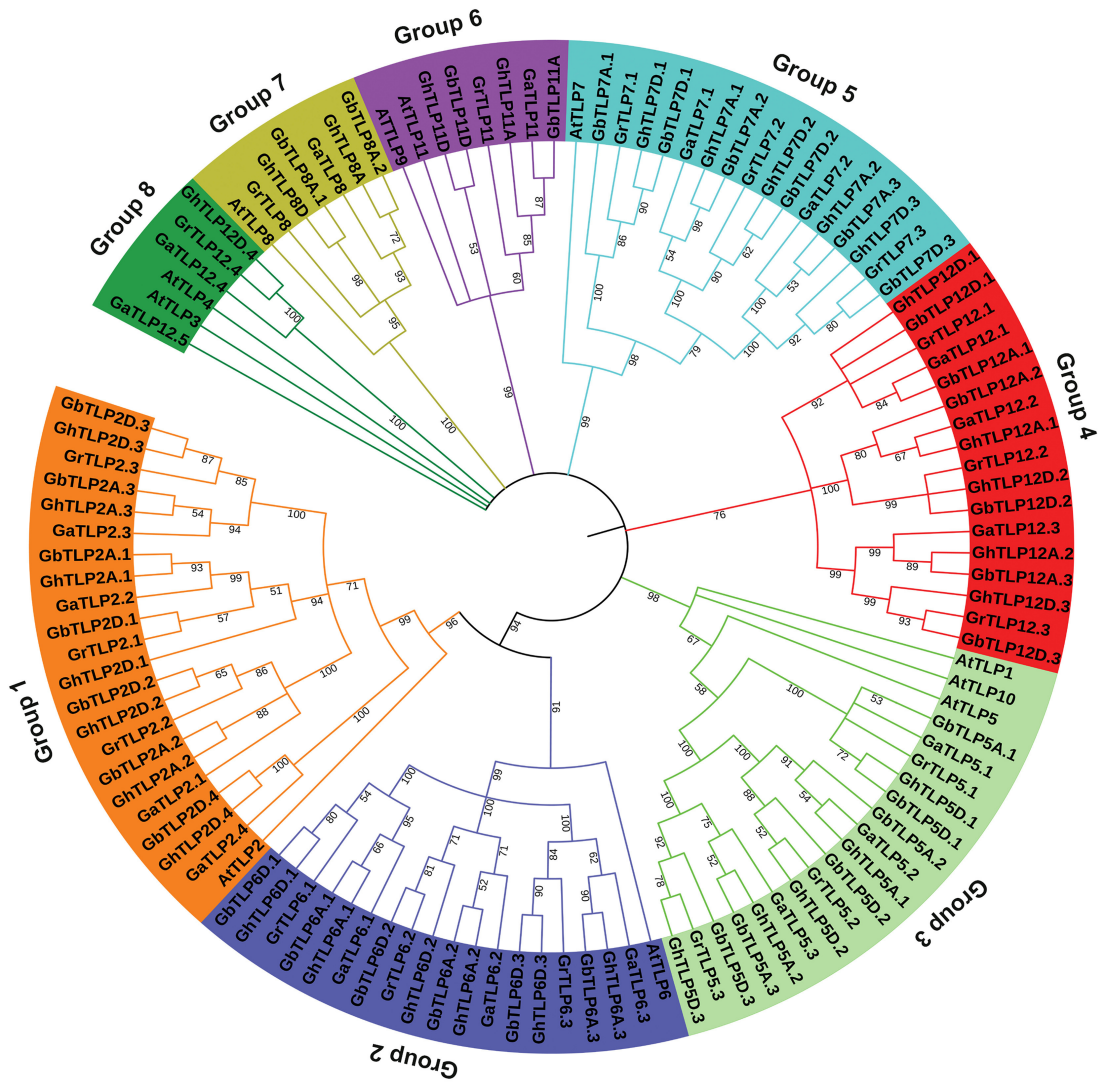
Phylogenetic relationship of *Gossypium* tubby-like proteins (TLPs) from *A. thaliana*. The phylogenetic ML tree was built, using the amino acid sequence with 1,000 bootstrap value with MEGA 7.0 software. At, *A. thaliana*; Ga, *Gossypium arboretum*; Gr, *Gossypium raimondii*; Gh, *Gossypium hirsutum*; Gb, *Gossypium barbadense*.

The multiple sequence alignment (MSA) of all cotton TLP genes showed a highly conserved C-terminal tubby domain and F-box at the C-terminal (Supplementary Figure 2). To determine the evolutionary relationship between TLP proteins of cotton and *A. thaliana*, MSA of 105 identified cotton TLPs with 11 *A. thaliana* TLPs was carried out. Furthermore, a phylogenetic tree was constructed, using the maximum likelihood tree (ML) method. On the basis of phylogenetic relationships, cotton *TLP* genes were clustered into eight major groups (Groups 1–8), each containing 21, 18, 17, 17, 16, 6, 6, and 4 TLPs, respectively (Figure 1).

**Figure 2:**
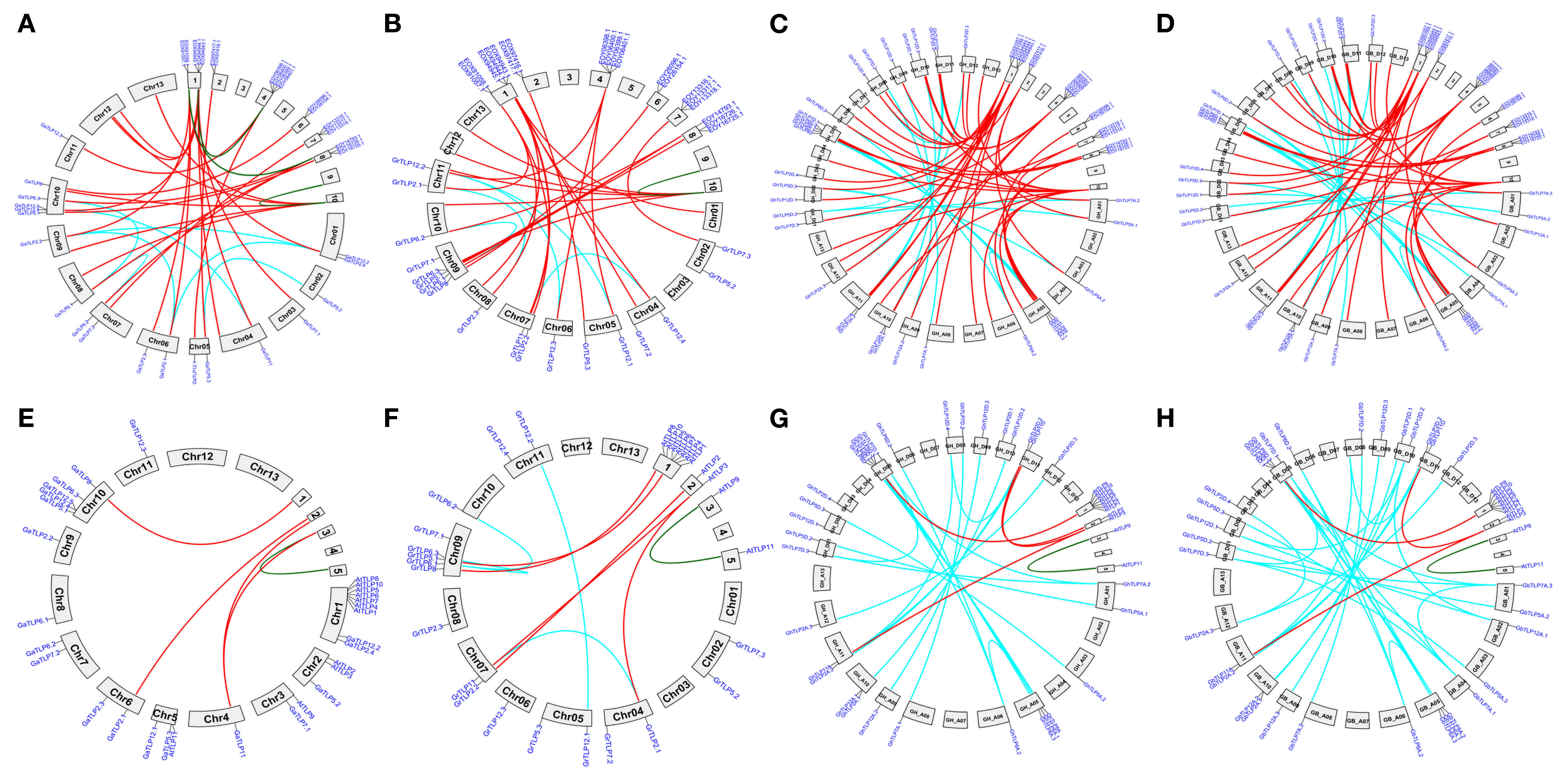
Synteny analysis of cotton *TLP* genes with *T. cacao* (Malvaceae) and *A. thaliana* (Brassicaceae) genomes. A syntenic representation of the identified *TLP* genes between **(A)** *G. arboreum* (Chr01 to Chr13) vs. *T. cacao* **(B)** *G. raimondii* (Chr01 to Chr13) vs. *T. cacao* **(C)** *G. hirsutum* (At1 to At13 and Dt1 to Dt13) vs. *T. cacao* (1–10) **(D)** *G. barbadense* (At1 to At13 and Dt1 to Dt13) vs. *T. cacao* (1–10) **(E)** *G. arboreum* (Chr01 to Chr13) vs. *A. thaliana* **(F)** *G. raimondii* (Chr01 to Chr13) vs. *A. thaliana* **(G)** *G. hirsutum* (At1 to At13 and Dt1 to Dt13) vs. *A. thaliana* **(H)** *G. barbadense* (At1 to At13 and Dt1 to Dt13) vs. *A. thaliana* chromosomes. Red lines indicate duplicated orthologous *TLP* genes, and cyan and green lines show the paralogous *TLPs* genes in *G. hirsutum, G. raimondii, G. arboreum, T. cacao*, and *A. thaliana*.

The majority of the cotton TLPs were found to be clustered with *A. thaliana TLPs*, the exception being groups 4 and 8 cotton *TLPs* (*GaTLPs12, GrTLPs12, GhTLPs12A, GhTLPs12D, GbTLPs12A*, and *GbTLPs12D*). To gain insight into the groups 4 and 8 *TLPs*, a phylogenetic tree of these *TLPs* with other eudicots (Ranunculaceae, Brassicaceae, Caricaceae, Cucurbitaceae, Rutaceae, Myrtaceae, Rosaceae, Fabaceae, Euphorbiaceae, Scrophulariaceae, Salicaceae, Solanaceae, Malvaceae, and Vitaceae) was generated. Also, synteny analysis of cotton *TLPs* with those from *A. thaliana* (Brassicaceae) and *T. cacao* (Malvaceae) was carried out. This revealed that groups 4 and 8 *TLPs* were greatly conserved among *G. arboreum* (A genome), *G. raimondii* (D genome), *G. hirsutum* (At and Dt sub-genomes), *G. barbadense* (At and Dt sub-genomes), and *T. cacao* genomes. The groups 4 and 8 cotton *TLP* genes were conserved among closely related species with *Gossypium* (Byng et al., 2016). On the other hand, no conserved homologs were found in Brassicaceae (Figure 2, Supplementary Figure 3, and Supplementary Table 2). The phylogenetic tree of groups 4 and 8 cotton *TLP* gene family members (*GaTLPs12, GrTLPs12, GhTLPs12A, GhTLPs12D, GbTLPs12A*, and *GbTLPs12D*) also revealed that groups 4 and 8 cotton *TLP* genes were not clustered with Brassicaceae family members (Supplementary Figure 3). This finding showed that, after the divergence from the common ancestor, the groups 4 and 8 cotton *TLP* homologs might have been evicted from the Brassicaceae family.

### Phylogenetic Tree, Encoded Protein Motifs, and Gene Structure Study of *Gossypium TLP* Genes

**Figure 3:**
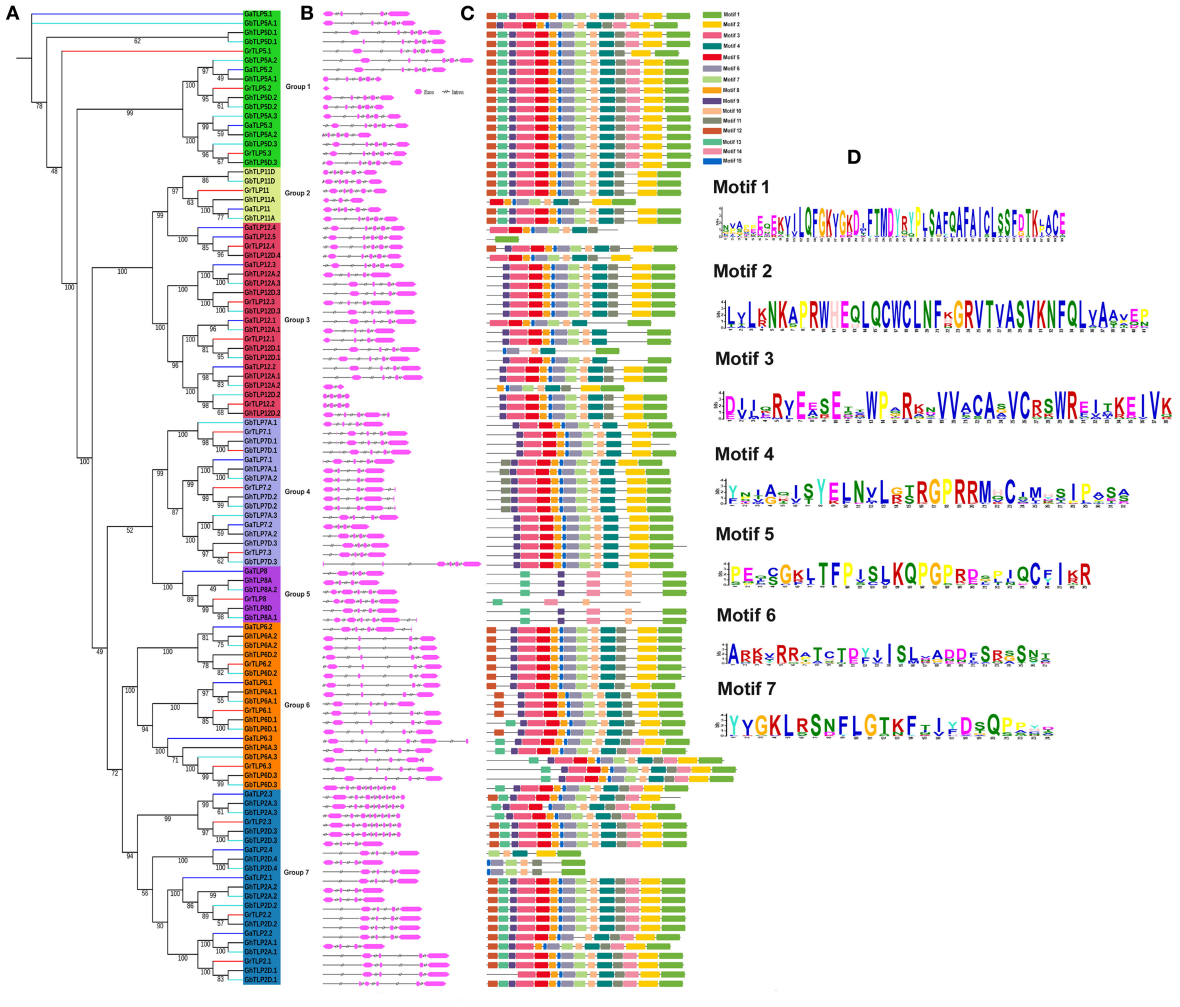
Phylogenetic tree, gene structure, and conserved protein motifs analysis of *TLPs* in *G. arboreum, G. raimondii, G. hirsutum*, and *G. barbadense*. **(A)** Phylogenetic tree of *G. arboreum, G. raimondii, G. hirsutum*, and *G. barbadense TLPs* built with the ML (maximum likelihood) method and using 1,000 bootstrap values. Different colors of lines denoted the different species of *Gossypium* (Blue, *G. arboreum*; Red, *G. raimondii*; Black, *G. hirsutum*; and cyan, *G. barbadense*). Groups 1–7 distributed in green, yellow, red, royal, violet, orange, and sky blue colors, respectively. **(B)** Showing the exon-intron organization of *TLPs* genes in *Gossypium* (Pink boxes, exon, and black lines, introns). **(C)** Identified conserved protein motifs in the *Gossypium TLPs*, and each motif is indicated with a specific color. **(D)** The logos are given for functionally annotated motifs only, where the heights of logo depict the degree of conservation of amino acid within the motif. The motifs order corresponds to their positions in protein sequence. Motif descriptions are given in Supplementary Table 3.

The evolutionary associations among the *Gossypium TLPs* were deduced by building a separate phylogenetic tree, using the ML method with 1,000 bootstraps value. On the basis of the topology of the tree, paralogous nodes, organization of exon–intron, and conservation of motifs, the cotton *TLP* genes were categorized into seven groups with higher bootstrap value. The proteins in each group had a high identity (>70%) among orthologous members but differed considerably from the members of the other groups, suggesting a divergent evolution from a common ancestor or origin from gene duplication events (Figure 3A). To determine the consistency of the exon-intron pattern in the phylogenetic groups, a gene structure comparison of the cotton *TLPs* was carried out. Intron number varied from 3 to 8 (*GaTLPs*), 0 to 8 (*GrTLPs*), 2 to 8 (*GhTLPs*), and 1 to 8 (*GbTLPs*) (Figure 3B and Table 1). The majority of the cotton *TLP* genes within the same group showed a similar pattern of exon-intron distribution. To study the conserved motif organization in TLP proteins, the MEME tool was employed for the analysis followed by annotation through InterProScan. A total of 15 conserved motifs were identified in the cotton TLP proteins. Only seven of these, motifs 1–7 (with the exception of motif 3), were found to form parts of the tubby domains. Motif 3 was annotated as the F-box domain (Figure 3D and Supplementary Table 3). Motif 1 was found in all cotton TLP proteins, except GaTLP12.4, GhTLP12D.4, and GhTLP7D.1. The majority of the cotton *TLPs* with close evolutionary relatives had similar motif composition and were assumed to have a similar function (Figure 3C).

### Chromosomal Location and Gene Duplication Events of *Gossypium TLP* Genes

**Figure 4:**
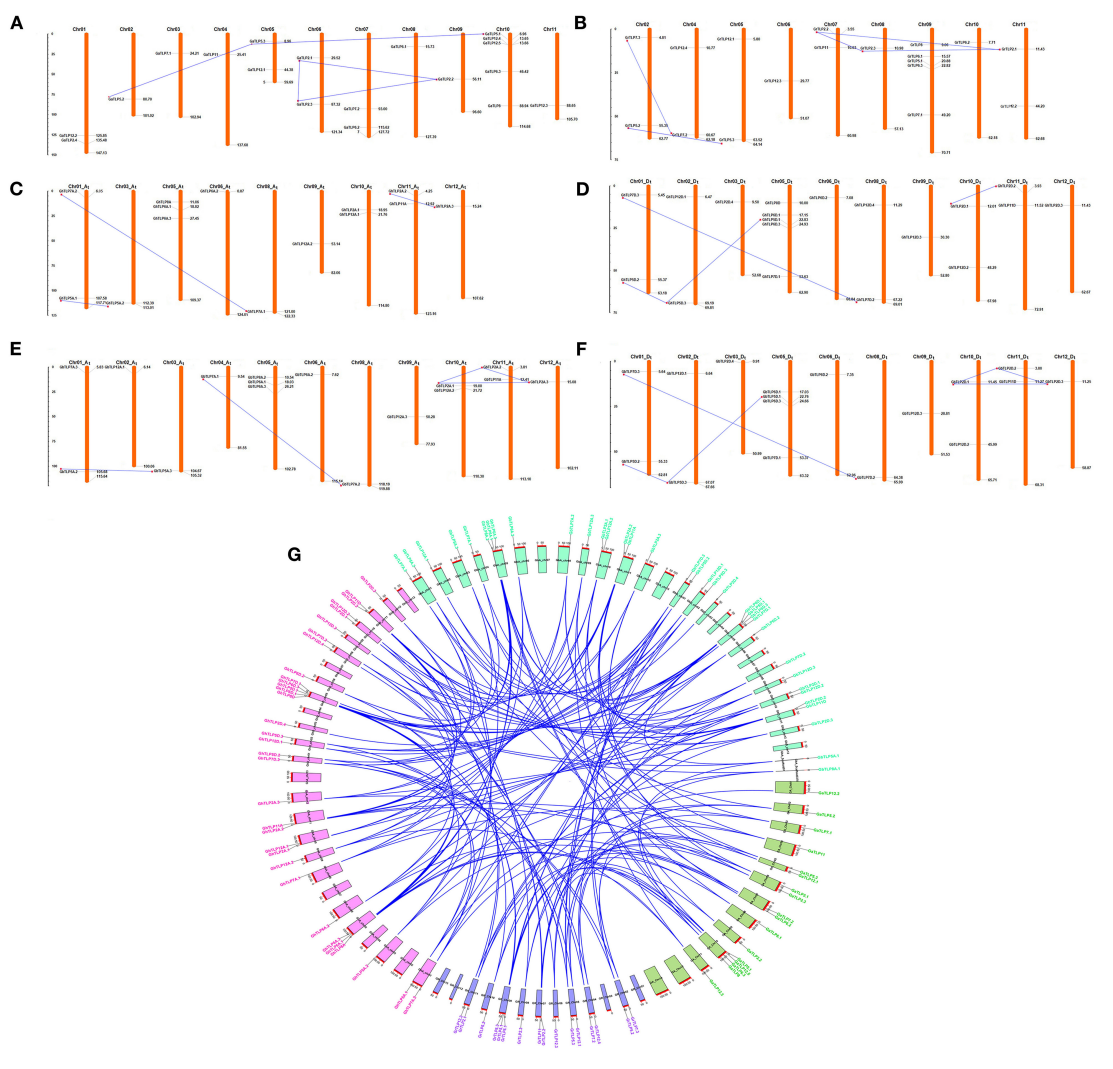
Chromosomal arrangement and paralogous gene duplication of *TLP* genes on the four genomes of *Gossypium*. A physical map of chromosomes demonstrates the position of *TLP* genes on A, D, and AD genomes, respectively. The paralogous *TLP* genes (segmental gene duplication) linked with blue lines. The locations of each *TLP* genes are represented through the horizontal gray lines. The numbers of chromosomes are showed at the top. **(A,B)** Represent the distribution of *TLP* genes in *G. arboreum* and *G. raimondii* genomes, **(C,D)** showed the arrangement of *TLP* genes in *G. hirsutum* genome, and **(E,F)** represented the *TLP* genes in *G. barbad*ense genome, respectively. The scale is in mega bases (Mb). **(G)** The orthologous *TLP* duplicated genes pairs among four cotton genomes (*G. arboreum, G. raimondii, G. hirsutum*, and *G. barbadense*) were also shown with blue lines, and CIRCOS was used to visualize this plot. The gene name and the chromosome of *G. arboreum, G. raimondii, G. hirsutum*, and *G. barbadense* were shown with green, violet, pink, and cyan colors, respectively.

To identify the chromosomal localization of *GaTLP, GrTLP, GhTLP*, and *GbTLP* genes in the cotton genome, the BLASTN search was performed. *GaTLP* genes were distributed on chromosomes 1–11 (Figure 4A), *GrTLP* genes were localized across chromosomes 2 and 4–11 (Figure 4B), *GhTLP* genes were located on At chromosomes 1, 3, 5, 6, 8–12 and on Dt chromosomes 1, 2, 3, 5, 6, 8–12 with 14 and 19 genes, respectively (Figures 4C,D), *GbTLP* genes were distributed on At chromosomes 1–6, 8–12, and Dt chromosomes 1, 2, 3, 5, 6, 8–12 with 16 and 17 genes, respectively (Figures 4E,F). Given the expansion of the number of cotton *TLP* genes, gene duplication events were next studied. High amino acid sequence similarities were detected among protein encoded by TLP genes, as five pairs of paralogous *TLP* genes were identified in diploid cotton (*G. arboreum* and *G. raimondii*) (Figures 4A,B), while seven pairs of the paralogous genes were determined in *G. hirsutum* (At and Dt sub-genomes) and 12 in *G. barbadense* (At and Dt sub-genomes) (Figures 4C–F). These paralogous *TLP* gene pairs existed in the same group, and most of them showed >70% sequence similarities between the proteins encoded by these *TLP* gene pairs. Except for the *GaTLP2.1/GaTLP2.3* gene pair, which was tandemly arranged, all other paralogous gene pairs were placed on distinct chromosomes, providing evidence that the expansion of the cotton the *TLP* family was mainly due to segmental duplication, not tandem duplication. In *G. arboreum*, four segmental gene duplications (*GaTLP5.2/5.3, GaTLP2.1/2.2, GaTLP2.2/2.3*, and *GaTLP5.1/5.3*) and one tandem duplication (*GaTLP2.1/2.3*) were occurred from 15.17 to 50.85 MYA. While five segmental duplications (*GrTLP5.2/5.3, GrTLP2.1/2.3, GrTLP2.2/2.3, GrTLP2.2/2.1*, and *GrTLP7.3/7.2*) were found in *G. raimondii* from 11.95 to 18.88 MYA (Table 2). In *G. hirsutum*, seven segmental gene duplications (*GhTLP5A.1/5A.2, GhTLP2A.2/2A.3, GhTLP7A.2/7A.1, GhTLP5D.2/5D.3, GhTLP5D.1/5D.3, GhTLP2D.1/2D.2*, and *GhTLP7D.3/7D.2*) occurred in At and Dt subgenomes from 14.77 to 53.60 MYA and 12 segmental duplication (*GbTLP8A.1/8A.2, GbTLP5A.2/5A.3, GbTLP2A.1/2A.3, GbTLP2A.1/2A.2, GbTLP2A.2/2A.3, GbTLP7A.2/7A.1, GbTLP5D.2/5D.3, GbTLP2D.1/2D.3, GbTLP2D.1/2D.2, GbTLP2D.2/2D.3, GbTLP7D.3/7D.2*, and *GbTLP5D.1/5D.3* occurred in At and Dt subgenomes of *G. barbadense* from 0.85 to 52.6 MYA (Table 2). Most of the paralogous gene pairs showed recent duplication events (13–20 MYA) (Li et al., 2014). The non-synonymous and synonymous substitution ratios (*Ka/Ks* ratios) for the duplicated *Gossypium TLP* gene pairs were consistently <1 (Table 2). Therefore, duplicated cotton *TLP* genes experienced intense purifying selection, which contributes to conserving their functions and reveals that not much diversion had taken place during the course of evolution (Gabaldon and Koonin, 2013). The orthologous gene pair, having <90% sequence identity in cDNA, and amino acid sequence were analyzed further for evolutionary studies (Supplementary Table 4). The selection pressure and the potential function of *Gossypium* TLPs were examined by computing the *Ka, Ks*, and *Ka/Ks* ratios among orthologous (A vs. D, At vs. A, Dt vs. D, At vs. At, and Dt vs. Dt) and within the homeologs (At vs. Dt). Interestingly, the *Ka* value of cotton orthologous *TLP2* (*GaTLP2/GrTLP2*), *TLP5* (*GaTLP5/GrTLP5*), *TLP6* (*GaTLP6/GrTLP6*), *TLP7* (*GaTLP7/GrTLP7*), and *TLP12* (groups 4 and 8 *TLPs*) (*GaTLP12/GrTLP12, GhTLP12A/GhTLP12D*, and *GbTLP12A/GbTLP12D*) genes were greater in inter-genomes (A vs. D and At vs. Dt) in comparison to other orthologous *TLP* gene pairs, suggesting that these pairs experienced faster evolution. Subsequently, during the course of evolution, orthologous *TLP* gene pairs often retain their corresponding function in different species (Gabaldon and Koonin, 2013). A total of 153 out of 172 orthologous *TLP* gene pairs have a *Ka/Ks* ratio <1, and the rest 16 have *Ka/Ks* >1 in both diploid and allotetraploid species, indicating a greater number of the *TLP* orthologous genes pairs experienced purifying selection pressure, and some of them experienced Darwinian selection pressure (Figure 4G and Supplementary Table 5). The *Ka/Ks* values of *TLP2, 5, 6, 7, 8, 11*, and *12* were higher in A vs. D, At vs. Dt, and Dt vs. D. Therefore, these *TLPs* experienced greater evolutionary pressure in diploid as well as in allotetraploid cotton and might have evolved rapidly in D subgenome as compared with A subgenome.

**Table 2:** The date of duplication and Ka/Ks ratios for duplicate *TLP* genes in *G. arboreum, G. raimondii, G. barbadense*, and *G. hirsutum*.

**Duplicated TLP gene1**	**Duplicated TLP gene2**	**Ka**	**Ks**	**Ka/Ks**	**Date (MYA) T = Ks/2λ**	**Selective pressure**	**Duplicate type**
GaTLP5.2	GaTLP5.3	0.0463	0.6175	0.0749	20.58	Purifying selection	Segmental
GaTLP2.1	GaTLP2.3	0.126	0.4551	0.277	15.17	Purifying selection	Tandem
GaTLP2.1	GaTLP2.2	0.0739	0.5349	0.1382	17.83	Purifying selection	Segmental
GaTLP2.2	GaTLP2.3	0.1161	0.5838	0.1989	19.46	Purifying selection	Segmental
GaTLP5.1	GaTLP5.3	0.0989	1.5263	0.0648	50.87	Purifying selection	Segmental
GrTLP5.2	GrTLP5.3	0.0476	0.5625	0.0847	18.75	Purifying selection	Segmental
GrTLP2.1	GrTLP2.3	0.0702	0.4551	0.1542	15.17	Purifying selection	Segmental
GrTLP2.2	GrTLP2.3	0.0802	0.3587	0.2235	11.95	Purifying selection	Segmental
GrTLP2.2	GrTLP2.1	0.0703	0.5049	0.1393	16.83	Purifying selection	Segmental
GrTLP7.3	GrTLP7.2	0.14	0.5666	0.2471	18.88	Purifying selection	Segmental
GhTLP5A.1	GhTLP5A.2	0.0489	0.602	0.0813	20.06	Purifying selection	Segmental
GhTLP2A.2	GhTLP2A.3	0.1161	0.4431	0.262	14.77	Purifying selection	Segmental
GhTLP7A.2	GhTLP7A.1	0.1516	0.6655	0.2278	22.18	Purifying selection	Segmental
GhTLP5D.2	GhTLP5D.3	0.0476	0.556	0.0856	18.53	Purifying selection	Segmental
GhTLP5D.1	GhTLP5D.3	0.0955	1.608	0.0594	53.6	Purifying selection	Segmental
GhTLP2D.1	GhTLP2D.2	0.1478	0.6154	0.2401	20.51	Purifying selection	Segmental
GhTLP7D.3	GhTLP7D.2	0.1552	0.58	0.2676	19.33	Purifying selection	Segmental
GbTLP8A.1	GbTLP8A.2	0.0079	0.0257	0.3079	0.85	Purifying selection	Segmental
GbTLP5A.2	GbTLP5A.3	0.0489	0.6016	0.0812	20.05	Purifying selection	Segmental
GbTLP2A.1	GbTLP2A.3	0.0726	0.5169	0.1404	17.23	Purifying selection	Segmental
GbTLP2A.1	GbTLP2A.2	0.0728	0.5132	0.1419	17.1	Purifying selection	Segmental
GbTLP2A.2	GbTLP2A.3	0.0854	0.3875	0.2204	12.91	Purifying selection	Segmental
GbTLP7A.2	GbTLP7A.1	0.1345	0.5374	0.2503	17.91	Purifying selection	Segmental
GbTLP5D.2	GbTLP5D.3	0.0498	0.5617	0.0886	18.72	Purifying selection	Segmental
GbTLP2D.1	GbTLP2D.3	0.0648	0.4543	0.1426	15.14	Purifying selection	Segmental
GbTLP2D.1	GbTLP2D.2	0.0703	0.4949	0.142	16.49	Purifying selection	Segmental
GbTLP2D.2	GbTLP2D.3	0.0786	0.3675	0.214	12.25	Purifying selection	Segmental
GbTLP7D.3	GbTLP7D.2	0.1445	0.573	0.2522	19.1	Purifying selection	Segmental
GbTLP5D.1	GbTLP5D.3	0.1004	1.578	0.0636	52.6	Purifying selection	Segmental

### Effects of Salt and Drought Stress on the Expression Profiles of *GhTLP* Genes

**Figure 5:**
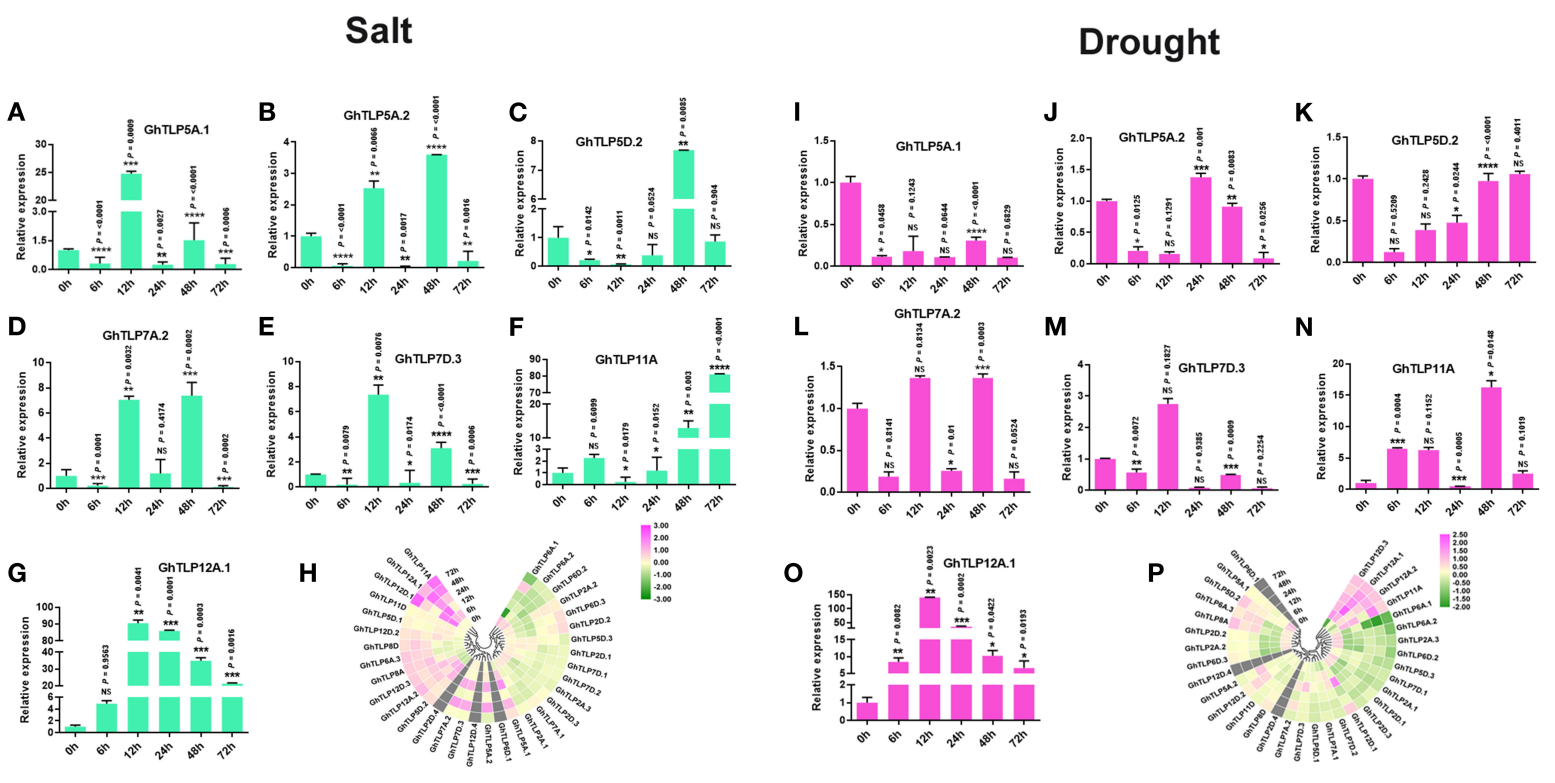
The expression pattern of the *TLP* gene family members in the *G. hirsutum* **(A–G,I–O)** qRT-PCR expression pattern of seven putative genes in normal (0 h), 6, 12, 24, 48, and 72 h of salt (300 MM) and drought (20% PEG solutions PEG8000) stress in cotton. Ubiquitin was used as the loading control. Three replicates were used for each experiment. The statistical analysis was performed, using two-tailed Student's *T*-tests. The data are plotted as means ± s.d. The error bars represent standard deviations. The asterisks indicate significant differences: *P < 0 0.1; **P < 0 0.01; ***P < 0 0.001; ****P < 0.0001. **(H,P)** Gene expression profiles of *TLP* genes under salt and drought condition in RNA-Seq, using Log2 (fold change) values.

It has been previously reported that *TLP* gene family members are expressed and regulated by several abiotic stresses (Lai et al., 2004; Wardhan et al., 2012; Bao et al., 2014). In view of these reports, we studied the involvement of cotton *TLP* genes in drought and salt stress conditions by analyzing transcriptomic data of leaf transcriptomes in response to drought and salt stress conditions. Thirty *GhTLP* genes exhibited differential expression, and twelve (*GhTLP5A.2, GhTLP5D.2, GhTLP6A.3, GhTLP7D.2, GhTLP7D.3, GhTLP8A, GhTLP11A, GhTLP12A.1, GhTLP12.2, GhTLP12D.1, GhTLP12D.2*, and *GhTLP12D.3*) showed significant higher expression during drought stress (Figure 5P) in transcriptome data. Furthermore, 17 *GhTLP* genes (*GhTLP2D.2, GhTLP6A.1, GhTLP6D.3, GhTLP5A.1, GhTLP5A.2, GhTLP5D.2, GhTLP6A.3, GhTLP7A.2, GhTLP7D.3, GhTLP8A, GhTLP8D, GhTLP11A, GhTLP12A.1, GhTLP12A.2, GhTLP12D.1, GhTLP12D.2*, and *GhTLP12D.3*) demonstrated higher expression in salt stress condition (Figure 5H). The studies show that the *GhTLP* gene family members respond to different abiotic stresses such as drought and salt and may have a role in regulating stress responses of cotton against salt and drought.

Furthermore, to validate the transcriptome data of *GhTLPs* in response to drought and salt stresses, the qRT-PCR validation of seven putative genes (*GhTLP5A.1, GhTLP5A.2, GhTLP5D.2, GhTLP7A.2, GhTLP7D.3, GhTLP11A*, and *GhTLP12A.1*) was carried out. The corresponding primers are listed in Table 3. The expression of seven *GhTLP* genes was significantly upregulated in salt-stressed plants, where the majority of the *GhTLPs* showed responses at 12 and 48 h (Figures 5A–G), while *GhTLP11A* and *GhTLP12A.1* showed highest responses in terms of fold change (FC) (>80 and >90) compared with control (Figures 5F,G). Similarly, six out of seven *GhTLP* genes were upregulated in drought-stressed plants, where *GhTLP5A.2* showed responses at 24 h (Figure 5J), *GhTLP5D.2* at 72 h (Figure 5K), *GhTLP7A.2* at 12 and 48 h (Figure 5L), *GhTLP7D.3* at 12 h (Figure 5M), *GhTLP11A* at 6, 12, 48, and 72 h (Figure 5N), but no expression was detected in *GhTLP5A.1* at any time scale (Figure 5I), while *GhTLP12A.1* showed highest response in terms of fold change (>140) in comparison to the control (Figure 5O). Altogether, the differential responses of *GhTLP* gene family members to salt and drought stresses suggest that *GhTLP* genes may function to combat abiotic stresses in cotton. Still, further studies are required to clone the significantly higher expressed genes to establish the role of individual *TLP* genes in cotton for salt and drought stress resistance.

**Table 3:** A list of primers used for validation in qRT-PCR.

	**Forward primer (5′-3′)**	**Reverse primer (5′-3′)**
GhTLP5A.1	TAGGCGGAGTTTTGATGTTAGATTG	ATTACAAGTGGCTCATCATGCAGAT
GhTLP5A.2	TCTCTAAATCTCTTGACCACTCTGTTGA	TTTTCCCGTCCTCATCATCATAA
GhTLP5D.2	TTTCAAGATCAAGCAGCAGTTACAT	CAGGCTGGGTATCGTATATTATGAATT
GhTLP7A.2	TTGCGTATGTAAGAAGTGGAGAGAA	AGTGATTTTGCCGCTATTTTGAG
GhTLP7D.3	TTGCGTATGTAAGAAGTGGAGAGAA	AAGTGATTTTGCCGCTATTTTGA
GhTLP11A	ATCAAAATCAACCCGTTCAGAGA	TCCTCAATACTAGCATTCCATCTTTCT
GhTLP12A.1	TATCAATCAACCCCAACTAGCTTTC	TCCACCATTCTTTTGATCAGATACA

### Co-expression Network and Pathways Analysis of Highly Expressed *GhTLP11A* and *GhTLP12A.1* Genes at Different Time Intervals Under Drought and Salt Stress Condition

The highly expressed genes (*GhTLP11A* and *GhTLP12A.1*) were further selected for co-expression network and pathways study. Using FPKM values, the co-expressed genes with *GhTLP11A* and *GhTLP12A.1* were explored. We identified positively co-expressed genes (PCoEGs) (2 and 4) and negatively co-expressed genes (NCoEGs) (5 and 14) in salt and drought stress with *GhTLP11A* (Supplementary Figure 4 and Supplementary Tables 6, 7). Similarly, PCoEGs (13 and 48) and NCoEGs (9 and 24) were determined with *GhTLP12A.1* in salt and drought stress (Supplementary Figure 4 and Supplementary Tables 6, 7).

PCoEGs and NCoEGs of *GhTLP11A* and *GhTLP12A.1* were subjected for PageMan pathways analysis to understand the molecular and functional role of these genes. The pathway study revealed that NCoEGs of *GhTLP11A* showed higher expression of calcium signaling, and PCoEGs of *GhTLP11A* displayed higher expression of *AS2* (lateral organ boundaries DOMAIN family protein) (Ma et al., 2006) at all time points (0, 6, 12, 24, 48, and 72 h) under salt stress condition (Supplementary Figure 5A). Earlier studies demonstrated that the elevated calcium levels help to protect plants from salt stress *via SOS* (salt overly sensitive) with signal transduction (Seifikalhor et al., 2019). In *Medicagotruncatula*, lateral organ boundaries domain (*LBD1*), *Sorghum bicolorLBD*, and *Vitis viniferaLBD* genes were upregulated under salt stress condition (Ariel et al., 2010; Wang et al., 2010; Grimplet et al., 2017), suggesting its role in salt stress response. Moreover, PCoEGs of *GhTLP12A.1* showed higher expression of β-ketoacyl-CoA synthase (*KCS*), a key enzyme for the fatty acid elongation (Yang et al., 2020b), and NCoEGs of *GhTLP12A.1* also displayed upregulation of a *PHD-type* transcriptional regulator at all time points (0, 6, 12, 24, 48, and 72 h) in salt stress response (Supplementary Figure 5B). Results revealed that β-ketoacyl-CoA synthase improves salt tolerance in *A. thaliana* (Yang et al., 2020b), and a *PHD-type* transcriptional regulator also improves salt tolerance in transgenic *A. thaliana* (Wei et al., 2009). Additionally, NCoEGs of *GhTLP11A* demonstrated little higher expression of jasmonate hormone metabolism, abiotic stresses for 12 h while the *MYB* domain and the *G2-like* transcriptional regulator highly expressed at all time points (0, 6, 12, 24, 48, and 72 h) in drought stress response (Supplementary Figure 5C). Methyl jasmonate (MeJA) has been reported to get enhanced during drought stress (Wu et al., 2012) and causes stomatal closure to save the water in wheat and enhance the antioxidant ability under the drought stress condition (Ma C. et al., 2014). Moreover, *MYB* has been reported to play a crucial role in providing tolerance under drought stress in *A. thaliana* and cotton (Zhang et al., 2012; Chen et al., 2015). These results suggested the important roles in drought stress tolerance. Alteration of its expression might improve tolerance under drought stress in cotton. Furthermore, NCoEGs of *GhTLP12A.1* showed higher expression in cellulose synthase enzyme related to the cell wall; PCoEGs of *GhTLP12A.1* displayed significantly highly expressed in lipid metabolism-related enzymes, the *DOF* zinc finger family, and *MAP* (mitogen-activated protein) kinases, signaling at all time points (0, 6, 12, 24, 48, and 72 h) in drought stress response (Supplementary Figure 5D). An earlier report revealed the importance of cellulose synthase in drought stress *via* induction of gene expression in *A. thaliana* (Chen et al., 2005), and lipid metabolism also showed an important role in drought stress response in *A. thaliana* by decreasing the lipid content progressively (Gigon et al., 2004). Moreover, overexpressed the *DOF* zinc finger family provides resistance under drought stress in *Populustrichocarpa* (Wang H. et al., 2017), and *MAP* kinases signaling also enables to enhance the tolerance under drought stress *via* the transmission of definite stimuli and regulating the antioxidant defense system (Sinha et al., 2011).

### Potential miRNA Target Sites in *Gossypium* TLP Transcripts

A large number of transcripts are regulated by miRNAs in response to stresses, signal transduction, and in plant development (Witkos et al., 2011). To study whether the cotton *TLP* genes may be regulated by miRNAs, target sites for miRNA binding were analyzed in the identified cotton *TLP* genes through the plant small RNA target analysis server (psRNATarget). The miRBase database possesses 378 cotton miRNAs. For the identification of miRNA target sites, the cut-off threshold of 4 was set in the search parameter. We were able to identify target sites for 41 cotton miRNAs in 56 cotton TLP transcripts with an expectation score (e) varied from 0.5 to 4 (Supplementary Table 8). Only the miRNA/target site pairs with cut-off 3.5 were selected to reduce the false-positive identification. Later, 44 miRNAs from the 14 miRNA families, comprising the target sites among 28 cotton *TLP* genes, were considered reliable in terms of e ≤3.5 (Table 4). Although the majority of the cotton *TLP* genes contain target sites for a single miRNA, some genes, such as *GbTLP2D.1, GbTLP7D.1, GhTLP2D.1, GhTLP7D.1, GrTLP2.1*, and *GrTLP7.1*, have target sites for more than one miRNA. Target site accessibility was evaluated by estimating unpaired energy (UPE), an essential factor in the identification of targets. The UPE of the target sites varies from 7.274 (gra-miR7494b) to 24.749 (gra-miR7486d), where a lesser amount of energy illustrated the greater chance of interaction among a miRNA and target sites (Marin and Vanicek, 2011).

**Table 4:** The potential miRNA target sites in cotton TLP transcripts.

**miRNA Acc**.	**Target Acc**.	**Expectation**	**Target accessibility (UPE)**	**miRNA length**	**Target start**	**Target end**	**Alignment**	**Inhibition**	**Multiplicity**
gra-miR7494b	GaTLP6.3	2.5	8.074	23	107	129	: .:::::: :::::.:.:::	Cleavage	1
gra-miR1446	GaTLP2.3	3	21.224	21	436	457	::::: :::::::::::::	Cleavage	1
gra-miR1446	GaTLP2.1	3	20.546	21	436	457	::::: :::::::::::::	Cleavage	1
gra-miR7494b	GrTLP6.3	2.5	8.002	23	392	414	: .:::::: :::::.:.:::	Cleavage	2
gra-miR1446	GrTLP2.3	3	21.16	21	445	466	::::: :::::::::::::	Cleavage	1
ghr-miR399a	GrTLP7.1	3.5	15.488	21	496	516	::. ::::.::.::::::	Cleavage	1
ghr-miR399b	GrTLP7.1	3.5	15.488	21	496	516	::. ::::.::.::::::	Cleavage	1
ghr-miR399d	GrTLP7.1	3.5	15.488	21	496	516	:::: ::::.::. ::::::	Cleavage	1
ghr-miR399e	GrTLP7.1	3.5	15.488	21	496	516	::: ::::.::. ::::::	Cleavage	1
gra-miR1446	GrTLP2.2	3.5	23.875	21	436	457	::::: ::::::.::::::	Cleavage	1
gra-miR482c	GrTLP12.1	3.5	23.054	22	679	700	:::::::: ::::::::	Translation	1
gra-miR7486d	GrTLP7.3	3.5	24.749	21	694	713	::::::::: : .:::::::	Translation	1
gra-miR7504k	GrTLP2.1	3.5	17.772	24	1,081	1,104	::.::.::::::::::.	Cleavage	1
gra-miR7504l	GrTLP2.1	3.5	17.772	24	1,081	1,104	::.::.::::::::::.	Cleavage	1
gra-miR7504m	GrTLP2.1	3.5	17.772	24	1,081	1,104	::.::.::::::::::.	Cleavage	1
gra-miR7494b	GhTLP6D.3	2.5	10.398	23	392	414	: .:::::: :::::.:.:::	Cleavage	1
gra-miR1446	GhTLP2D.3	3	21.16	21	445	466	::::: :::::::::::::	Cleavage	1
gra-miR1446	GhTLP2A.2	3	20.546	21	436	457	::::: :::::::::::::	Cleavage	1
ghr-miR399a	GhTLP7D.1	3.5	15.775	21	496	516	::. ::::.::.::::::	Cleavage	1
ghr-miR399b	GhTLP7D.1	3.5	15.775	21	496	516	::. ::::.::.::::::	Cleavage	1
ghr-miR399d	GhTLP7D.1	3.5	15.775	21	496	516	:::: ::::.::. ::::::	Cleavage	1
ghr-miR399e	GhTLP7D.1	3.5	15.775	21	496	516	::: ::::.::. ::::::	Cleavage	1
ghr-miR7502	GhTLP6D.1	3.5	15.52	24	68	91	::. :::.:::: :::::::	Cleavage	1
gra-miR482c	GhTLP12D.1	3.5	22.507	22	358	379	:::::::: ::::::::	Translation	1
gra-miR7504k	GhTLP2D.1	3.5	17.772	24	1,093	1,116	::.::.::::::::::.	Cleavage	1
gra-miR7504l	GhTLP2D.1	3.5	17.772	24	1,093	1,116	::.::.::::::::::.	Cleavage	1
gra-miR7504m	GhTLP2D.1	3.5	17.772	24	1,093	1,116	::.::.::::::::::.	Cleavage	1
gra-miR8700	GhTLP7D.1	3.5	19.294	24	1,067	1,090	:::: ::: ::::: ::::	Translation	1
gra-miR8767c	GhTLP5D.1	3.5	15.286	21	1,158	1,178	:.::::.: :::::.:::	Translation	1
gra-miR7494b	GbTLP6D.3	2.5	10.515	23	107	129	: .:::::: :::::.:.:::	Cleavage	1
gra-miR7494b	GbTLP6A.3	2.5	7.274	23	314	336	: .:::::: :::::.:.:::	Cleavage	1
gra-miR1446	GbTLP2A.2	3	20.546	21	436	457	::::: :::::::::::::	Cleavage	1
gra-miR1446	GbTLP2D.3	3	21.16	21	445	466	::::: :::::::::::::	Cleavage	1
gra-miR1446	GbTLP2A.3	3	21.972	21	412	433	::::: :::::::::::::	Cleavage	1
ghr-miR399a	GbTLP7D.1	3.5	15.775	21	496	516	::. ::::.::.::::::	Cleavage	1
ghr-miR399b	GbTLP7D.1	3.5	15.775	21	496	516	::. ::::.::.::::::	Cleavage	1
ghr-miR399d	GbTLP7D.1	3.5	15.775	21	496	516	:::: ::::.::. ::::::	Cleavage	1
ghr-miR399e	GbTLP7D.1	3.5	15.775	21	496	516	::: ::::.::. ::::::	Cleavage	1
gra-miR482c	GbTLP12D.1	3.5	22.507	22	682	703	:::::::: ::::::::	Translation	1
gra-miR7486d	GbTLP7D.3	3.5	24.749	21	694	713	::::::::: : .:::::::	Translation	1
gra-miR7504k	GbTLP2D.1	3.5	17.772	24	1,081	1,104	::.::.::::::::::.	Cleavage	1
gra-miR7504l	GbTLP2D.1	3.5	17.772	24	1,081	1,104	::.::.::::::::::.	Cleavage	1
gra-miR7504m	GbTLP2D.1	3.5	17.772	24	1,081	1,104	::.::.::::::::::.	Cleavage	1
gra-miR8767c	GbTLP5D.1	3.5	15.286	21	1,158	1,178	:.::::.: :::::.:::	Translation	1

### *cis*-Regulatory Element Analysis of *GhTLP*s

**Figure 6:**
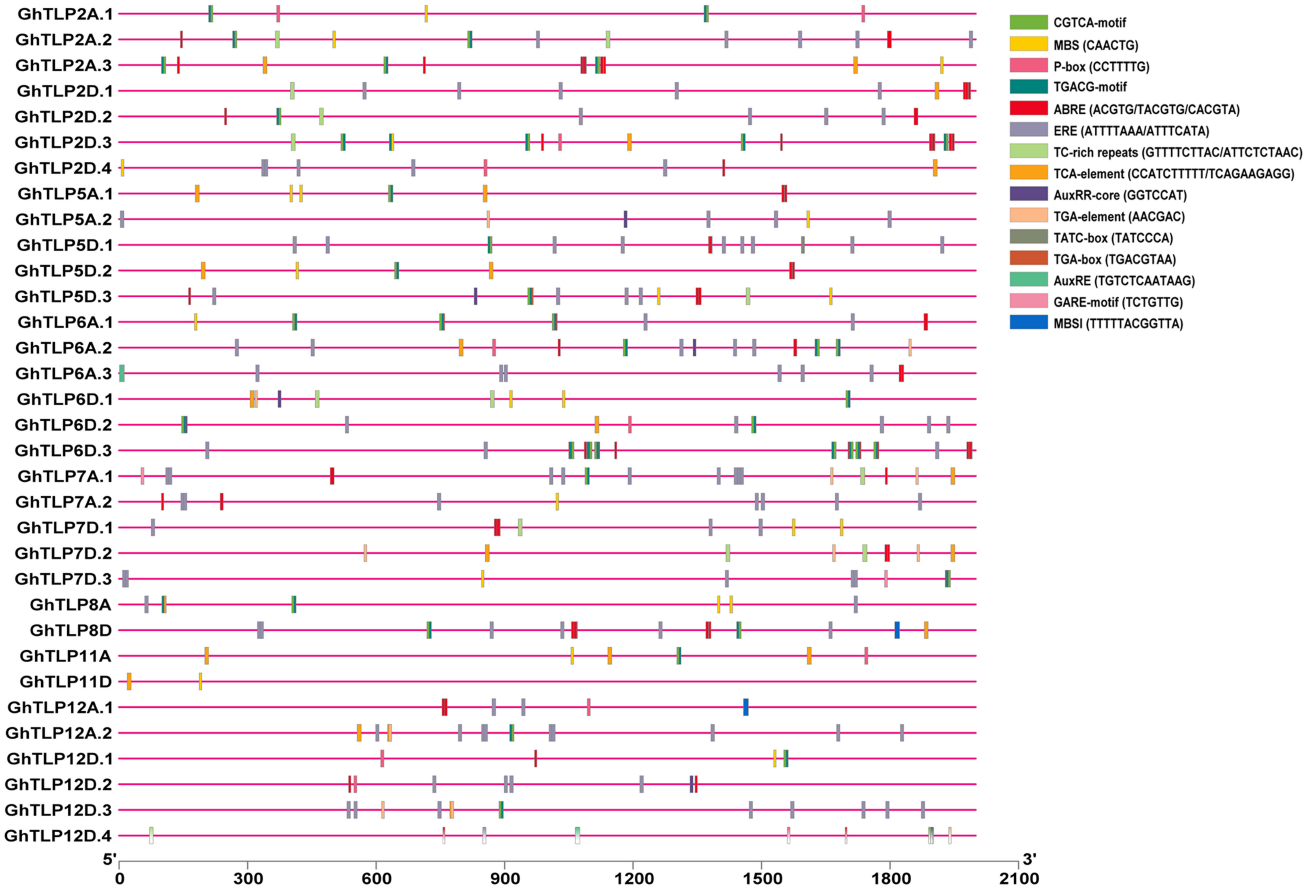
*cis*-regulatory elements analysis of *G. hirsutum TLP* genes. The micro-parts in diverse colors are the putative elements sequence.

The *cis*-regulatory elements are crucial to controlling the regulation of transcription in several stress conditions (Nakashima et al., 2009). To identify *cis*-regulatory elements that may govern *TLP* expression in cotton, a 2-Kb region upstream from the translational start site of *GhTLPs* was scanned for various *cis* elements. A total of 1,182 proximal elements were identified in 33 *GhTLPs* that included 737 for abiotic stresses, 343 for hormonal responses, 30 for biotic stresses and 72 elements for the other *cis*-regulatory elements (Figure 6 and Table 5). A higher number of *cis*-regulatory elements were identified in *GhTLP2D.2* (60), whereas the least number of *cis*-regulatory elements were detected in *GhTLP12A.1* (18) (Supplementary Table 9). In abiotic stress-responsive *cis*-regulatory elements, the majority of the elements were involved in light responses, followed by low temperature, flavonoid biosynthesis, defense, and stress (Supplementary Table 9). The *cis*-regulatory elements related to hormonal responses comprised auxin, salicylic acid (SA), abscisic acid, gibberellin, and methyl jasmonate-responsive elements (Supplementary Table 9). The SA-responsive TCA element was present in a higher number in *GhTLP11A*. The auxin-responsive AuxRR-core element, which has an important role in salt, as well as drought stress responses (Guo et al., 2018; Kang et al., 2018), was detected in *GhTLP5A.2, GhTLP5D.3*, and *GhTLP6A.2*. Ethylene-responsive elements (EREs), which provide defense against salt and drought stress conditions (Pei et al., 2017; Sharma et al., 2019), were also detected and present in a higher number in *GhTLP5D.2*. The presence of these *cis*-regulatory elements in *GhTLPs* hints at their potential roles in the regulation of gene expression in cotton growth and development as well as under various environmental conditions (Nawaz et al., 2014).

**Table 5:** Identified *cis*-regulatory elements in the *GhTLPs* genes.

	**Abiotic stress responsive elements**	**Biotic stress responsive elements**	**Hormone responsive elements**	**Others**
GhTLP2A.1	15		6	2
GhTLP2A.2	41		6	3
GhTLP2A.3	26	1	11	3
GhTLP2D.1	22		15	2
GhTLP2D.2	46	1	9	4
GhTLP2D.3	28		8	4
GhTLP2D.4	24	1	20	1
GhTLP5A.1	28	2	8	1
GhTLP5A.2	14		7	3
GhTLP5D.1	12	1	6	2
GhTLP5D.2	26	3	13	1
GhTLP5D.3	18		8	3
GhTLP6A.1	27	1	11	3
GhTLP6A.2	25	3	12	1
GhTLP6A.3	19		17	0
GhTLP6D.1	28		9	6
GhTLP6D.2	20	1	5	1
GhTLP6D.3	25	1	11	1
GhTLP7A.1	30		29	0
GhTLP7A.2	15	2	16	3
GhTLP7D.1	22	3	9	2
GhTLP7D.2	22		7	0
GhTLP7D.3	21	1	11	2
GhTLP8A	26	2	8	3
GhTLP8D	20		8	4
GhTLP11A	8		17	5
GhTLP11D	15		6	4
GhTLP12A.1	13		4	1
GhTLP12A.2	18		7	3
GhTLP12D.1	18	3	14	1
GhTLP12D.2	15	1	4	2
GhTLP12D.3	26	1	7	1
GhTLP12D.4	24	2	14	0
Total	737	30	343	72

### Three-Dimensional (3D) Structural Analysis of the Putative GhTLPs Tubby Domain

**Figure 7:**
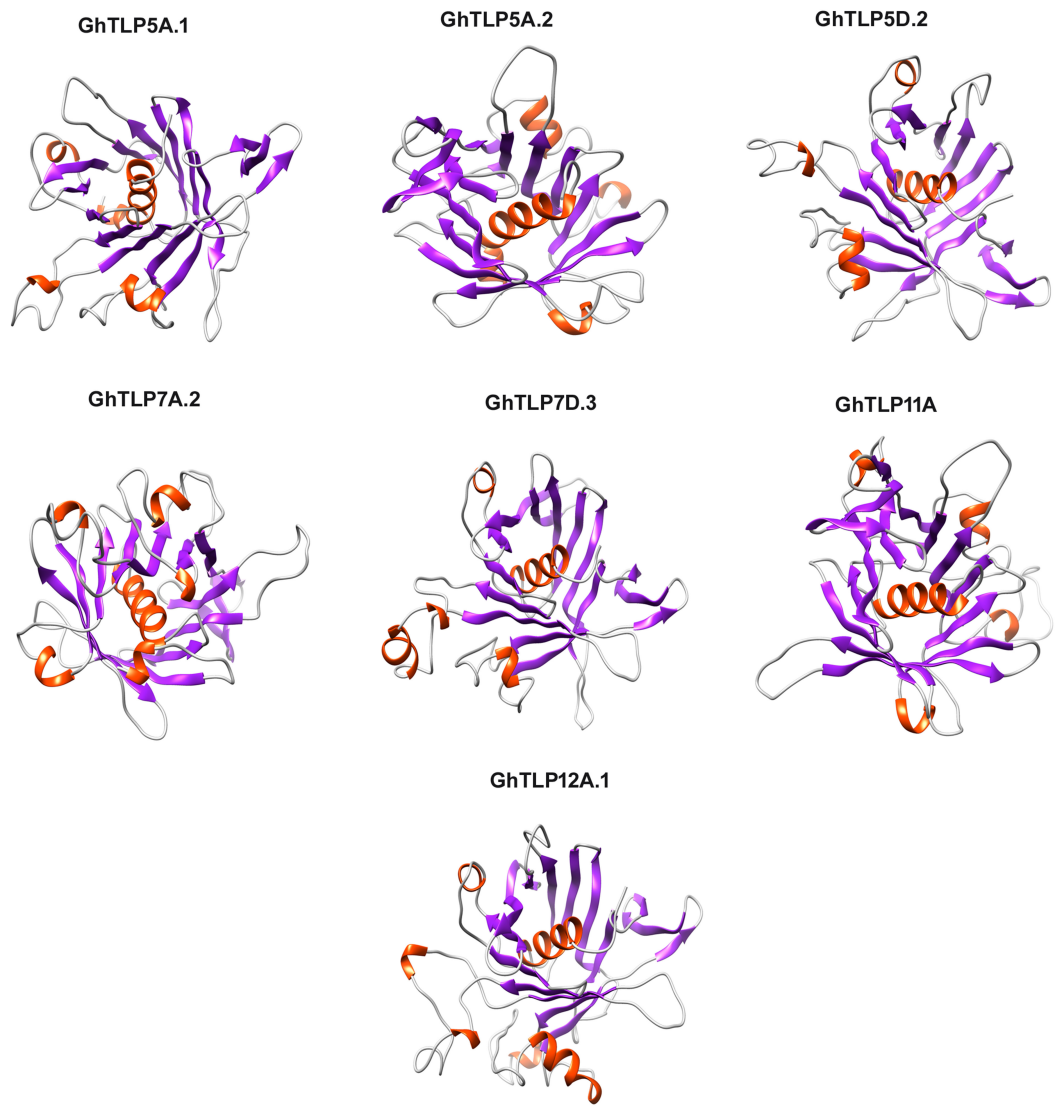
The homology 3D model of the GhTLPs tubby domain. The GhTLPs were selected for three-dimensional structure prediction and display with confidence level >99%. The alpha helix is shown with red and beta fold is shown with violet color.

The 3D structures of the GhTLPs tubby domain were determined through homology modeling of a central alpha-helix surrounded by a beta-barrel (Figure 7). All the putative GhTLPs have a typical tubby structure with a closed beta-barrel formed by 12 anti-parallel strands and a central alpha helix. While most GhTLPs contain five alpha-helices, GhTLP7A.2 and GhTLP12A.1 comprise six alpha-helices. These three-dimensional structural differences might lead to the functional diversification of different GhTLPs and suggest a slightly altered role for GhTLP7A.2 and GhTLP12A.1 as compared with the other GhTLP proteins. The higher transcript level of *GhTLP12A.1* during drought and salt stress conditions further supported this hypothesis.

## Discussion

The genus *Gossypium* includes ~45 diploid (*2n* = 2x = 26) and six tetraploid (*2n* = 4x = 52) species (Hawkins et al., 2006; Grover et al., 2015). *G. hirsutum* and *G. barbadense* are allotetraploids that have been arisen in the new world from interspecific hybridization among A-genome-like ancestral African species and D-genome-like American species (Chen et al., 2007). The closest relatives of the tetraploid progenitors are the A-genome species *G. herbaceum* (A1) and *G. arboreum* (A2) and the D-genome species, *G. raimondii* (D5) or *G. gossypioides* (D6) (Brubaker et al., 1999; Senchina et al., 2003). Approximately, 1–2 million years ago, polyploidization occurred, giving rise to allotetraploid species (Wendel and Cronn, 2003). *G. hirsutum* and *G. barbadense* are possibly the oldest main allopolyploid crops (Paterson et al., 2012; Chalhoub et al., 2014; Marcussen et al., 2014).

In our efforts to study the *TLP* family in cotton, we have identified a total of 105 cotton *TLP* genes in four *Gossypium* genomes (*G. arboreum, G. ramondii, G. hirsutum*, and *G. barbadense*), 19 *GaTLPs*, 18 *GrTLPs*, 33 *GhTLPs*, and 35 *GbTLPs* (Table 1 and Supplementary Table 1). The genome sizes of *G. arboreum* and *G. raimondii* are 1,746 and 885 Mb (Li et al., 2014), respectively, and, expectedly, *G. arboreum* had higher numbers of *TLP* genes as compared with *G. raimondii*. However, although the genome size of *G. hirsutum* (~2.30 Gb) (Hu et al., 2019) was about the same as *G. barbadense* genome size (~2.22 Gb) (Hu et al., 2019), *G. barbadense* had a greater number of *TLP* genes as compared with *G. hirsutum*. The higher number of the *TLP* gene family members in *G. barbadense* might be due to the whole genome duplication events (Zhang et al., 2015; Qiao et al., 2019) which facilitate diversification (Clark and Donoghue, 2018).

The domain study revealed that all the conserved cotton TLP proteins comprised the tubby domain at the C-terminal and F-box domain at the N- terminal end while some of the cotton TLPs lack the F-box (Supplementary Figure 1). This was also observed in *Arabidopsis thaliana*, indicating the functional role of TLP proteins lacking the F box (Lai et al., 2004). The phylogenetic analysis of cotton *TLP*s and *A. thaliana* protein sequences grouped the cotton TLP proteins into eight major groups (Groups 1–8). However, groups 4 and 8 cotton *TLP* genes were not clustered with any of *A. thaliana* genes (Figure 1). Further analysis of groups 4 and 8 cotton *TLP* genes with other eudicots showed the loss of these genes from the brassicaceae family only. The orthologous gene pair analysis of the cotton *TLP12* genes (Groups 4 and 8) with *A. thaliana* and *T. cacao* (closest relative of *Gossypium*). The outcomes of synteny analysis showed that cotton the *TLP12* gene family members (Groups 4 and 8) have orthologous duplicated genes in *T. cacoa* (Figures 2A–D) while no orthologous duplicated genes were detected in *A. thaliana* (Figures 2E–H). Therefore, it may be hypothesized that groups four and eight cotton *TLP* genes (*TLP12*) were the consequence of recent species-specific duplication events that led to independent functional diversification. Groups four and eight *TLPs* orthologous pairs experienced faster evolution as compared with the other *TLP* gene family members, indicating their functional divergence in *Gossypium*, proposing that groups four and eight *TLPs* might have a specific function in cotton species. The identified orthologous *TLP12* gene pairs in *G. hirsutum* and *G. barbadense* are approximately double in comparison to *G. arboreum* and *G. raimondii*, respectively, showing the effect of polyploidy. This leads to more orthologous gene pairs in *GhTLP12* and *GbTLP12* genes than *GaTLP12* and *GrTLP12* genes (Qanmber et al., 2019a).

The evolutionary analysis within the *Gossypium TLP* genes showed most of them were greatly conserved during evolution, showed introns of these genes were not lost during evolution, and, at the early expansion stages of evolution, these genes diverged, whereas, over evolutionary time, other genes lost their introns (Qanmber et al., 2019b), indicating that group specific genes may have similar functions. According to a previous report on gene structure, introns performed essential functions during the course of evolution in several plant species (Roy and Gilbert, 2006). During the early expansion period, there was more intron, which subsequently decreased over the passage of time (Roy and Penny, 2007). GrTLP5.2 comprises no intron in gene structure; lack of intron indicates that the *TLP* gene is advanced where introns were disappeared over the evolutionary time period (Qanmber et al., 2019a); this gene might have some conserved evolutionary function in cotton. These findings demonstrated more advanced species comprise fewer introns in their genomes (Roy and Gilbert, 2005). Higher number of introns led to new functions (Qanmber et al., 2019a). Moreover, several gene families comprise no intron or with fewer introns in their genes (Zhang et al., 2015; Qanmber et al., 2018). Insertions or deletions events participate in the structural differences of exon/intron that might be useful to calculate the evolutionary mechanisms (Lecharny et al., 2003). Introns are absent in some genes that might be due to a rapid evolution rate, whereas a greater number of introns comprising genes leads to a gain of function in evolution (Qanmber et al., 2019b). The loss or gain of genes through segmental duplication or incomplete sequencing of genomes is the major cause for *TLP* genes distribution in cotton (Qanmber et al., 2019b).

Chromosomal allocation studies demonstrated that cotton *TLP* genes expansion has arisen due to segmental duplication except for *GaTLP2.1/GaTLP2.3* (Figures 4A–F). The purifying selection probably excludes the deleterious loss-of-function mutations, refining functional alleles at both duplicate loci and fixing recent duplicate genes (Tanaka et al., 2009). All the identified paralogous cotton *TLP* gene pairs indicated the purifying selection (Table 2). The recent duplication events in *Gossypium TLPs* have had implicit ecological, morphological, and physiological diversification (Wendel and Cronn, 2003). The diploid genomes of *G. arboreum* and *G. raimondii* were diverged 2–13 MYA, and allotetraploid cotton (*G. hirsutum* and *G. barbadense*) was originated about 1–2 MYA (Li et al., 2014; Wang M. J. et al., 2017; Wang et al., 2019). The duplication time of *GaTLPs* (15.17–50.87 MYA), *GrTLPs* (11.95–18.88 MYA), *GhTLPs* (14.77–53.60 MYA), and *GbTLPs* (0.85–52.6 MYA) implied that duplication events in *Gossypium TLP* gene families were more ancient than that of both polyploid formation and divergence of diploid species. This duplication might facilitate the unique role of *TLP* genes in *Gossypium*, i.e., cotton stress responses. The average duplication time of *GaTLPs* and *GrTLPs* was around 24.78 and 16.31 MYA, which probably took place after their divergence from *T. cacoa* (33 MYA) (Li et al., 2014) and *A. thaliana* (93 MYA) (Ma et al., 2016); before the reunification of A and D diploid genomes that lead to allotetraploid cotton (Zhang et al., 2015; Wang et al., 2019) (Table 2). These observations suggested that *TLP2.2* and *TLP2.3* in *G. arboreum, G. raimondii*, and *G. barbadense* might have arisen from the same duplication event of cotton *TLP2.1* genes. All paralogous cotton *TLP* gene pairs except *GaTLP2.1/GaTLP2.3* experienced segmental duplication (Figures 4A–F). Here, both segmental and tandem duplication helped in the *TLP* gene family expansion, but segmental duplication might have some significant role in the expansion of the *TLP* gene family members (Liu et al., 2018; Qanmber et al., 2019a; Ali et al., 2020).

The orthologous gene pairs had the sequence identity >90% in cDNA and also in amino acid compositions (Supplementary Table 4), which were carried out for further evolutionary study. Among orthologous-duplicated pairs, the *Ka/Ks* values of *TLP2, TLP5, TLP6, TLP7, TLP8, TLP11*, and *TLP12* were higher in A vs. D, At vs. Dt, and Dt vs. D. The divergence analysis showed that cotton *TLPs* experienced greater evolutionary pressure in diploid as well as in allotetraploid cotton and might have evolved rapidly in D subgenome as compared with A subgenome (Supplementary Table 5). All the identified orthologous cotton *TLP* gene pairs show purifying selection (Figure 4G and Supplementary Table 5).

The *TLP* genes are known to play important roles in stress responses in various plant species (Lai et al., 2004; Liu, 2008; Xu et al., 2016). The transcriptome analysis data of *G. hirsutum* showed the high expression of *GhTLP* genes in salt and drought stresses. The expression analysis showed that *GhTLP5A.1* and *GhTLP5D.2* genes have a significantly higher relative expression in salt stress response, but not in drought stress; therefore, these genes might have a major role in salt-stress tolerance. Moreover, *GhTLP11A* and *GhTLP1*2A.1 showed higher expression in both salt and drought-stress responses. Therefore, these two genes might have an important role in salt and drought tolerance and could be appropriate targets for further manipulation to protect the cotton from salt and drought stress.

To further characterize the function of *GhTLP11A* and *GhTLP12A.1* genes, the co-expression network of these two genes (Supplementary Figure 4) was studied. This study revealed that PCoEGs of *GhTLP11A* comprised the lateral organ boundaries (LOB) domain (*LBD*), which were upregulated *via* ABA treatment in *Vitis vinifera* under salt-stress response (Grimplet et al., 2017). NCoEGs of *GhTLP11A* contained ABC transporter-like protein, actively involved in salt-stress recovery in *Populuseuphratica* (Gu et al., 2004) and calcium protein, which was considered as one of the important molecules in response to salinity (Seifikalhor et al., 2019), and, in a seedling of rice, Ca^2+^ induces antioxidant enzyme activity and retains cellular redox potential under salt stress (Rahman et al., 2016; Supplementary Tables 6, 7). In salt stress, PCoEGs of *GhTLP12A.1* comprised the *SANT/MYB* domain and the sugar-phosphate transporter domain. Sugarcane *MYB18*, containing the *SANT/MYB* DNA-binding domain, remarkably improved tolerance to salt stress (Shingote et al., 2015) and phosphate transporter *PHT4;6* of *A. thaliana* function in cell wall biosynthesis and protein N-glycosylation, which are crucial to salt tolerance (Cubero et al., 2009). NCoEGs of *GhTLP12A.1* contained a *FYVE/PHD-type* zinc finger and *MADS-box* in salt stress. *A. thaliana* RING/FYVE/PHD ZFP (*AtRZFP*) is found to bind with zinc and provides tolerance to salt stress (Zang et al., 2016), and *MADS-box* considered a positive regulator of salt-stress response *via* regulating the maintenance of ABA signaling, primary metabolism, detoxification, and ROS homeostasis through antioxidant enzymatic activities (Castelán-Muñoz et al., 2019; Supplementary Tables 6, 7). Results demonstrated that PCoEGs and NCoEGs of *GhTLP11A* and *GhTLP12A.1* genes might be crucial in salt-stress responses. In drought stress, PCoEGs of *GhTLP11A* comprised a protein kinase domain and haloacid dehalogenase-like hydrolase (*HAD hydrolase*). Calcium-dependent protein kinase may function as calcium sensors and have an important role in drought-stress response. In *A. thaliana*, calcium-dependent protein kinase 10 (*CPK10*) provides tolerance under drought stress *via* ABA and Ca^2+^-mediated stomatal regulation (Zou et al., 2010). *A. thaliana* trehalose-6-phosphate phosphatases (*AtTPPs*) encodes a protein in the *HAD hydrolase* superfamily that is involved in the biosynthesis of trehalose. Overexpressed *AtTPPF* leads to the accumulation of trehalose in response to drought stress and can increase the tolerance under drought stress (Lin et al., 2019). NCoEGs of *GhTLP11A* contained a B3 domain, which improves drought-stress tolerance *via* reducing the stomatal density and changed the shape of stomata in *Zea mays* (Liu Y. H. et al., 2015) and a late embryogenesis abundant (*LEA*) gene, whose higher expression provides tolerance under drought stress in upland cotton (Magwanga et al., 2018; Supplementary Table 7). PCoEGs of *GhTLP12A.1* comprised expansin and a *Dof-type* zinc finger in drought-stress response. Transgenic wheat expansin 2 (*TaEXPA2*) positively regulates tolerance under drought stress (Yang et al., 2020), and *Brassica rapa* expansin-like B1 (*BrEXLB1*) also associated with drought stress tolerance (Muthusamy et al., 2020), while the overexpressed DOF zinc finger family provides resistance under drought stress in *P. trichocarpa* (Wang H. et al., 2017). NCoEGs of *GhTLP12A.1* contained *UBA-like* superfamily and dirigent protein under drought stress response (Supplementary Table 7). In wheat, a UBA protein (*TaUBA*), a negative regulator of drought stress, might function *via* downregulating some stress responsive transcription factors (Li et al., 2015), and, in *Boeahygrometrica*, dirigent proteins provide a protective role under drought-stress response *via* changing the physical characters of lignin, which further affects the flexibility and mechanical strength of the plant cell wall (Wu et al., 2009). Taken together, our results showed that PCoEGs and NCoEGs of *GhTLP11A* and *GhTLP12A.1* genes might have a crucial role in drought-stress tolerance.

Moreover, we determined 41 miRNA target sites in 56 cotton *TLP* transcripts with an expectation score (E) varied from 0.5 to 4 (Supplementary Table 8). In this study, 15 miRNA families, comprising target sites in 28 cotton *TLP* genes, were detected (Table 4). An earlier report showed that some of the miRNA families were conserved among the plants, which displayed their function in the adaptation of plants to various stress responses (Jones-Rhoades and Bartel, 2004; Yuan et al., 2013). In *Vitis vinifera*, miR7494 has a prominent role in plants under abiotic stresses, and, in *Zea mays*, the expression of miR399 gets induced during abiotic stress response (Zhang et al., 2008; Kumar, 2014; Pagliarani et al., 2017; Snyman et al., 2017; Inal et al., 2020). These miRNAs that have been detected in this study are with lower UPE value (7.27–15.77) (Table 4). These outcomes suggested that cotton miRNAs might also be involved in abiotic stress responses to enhance drought- and salt-stress tolerance.

Moreover, *cis*-regulatory element analysis demonstrated that, among the selected putative genes for validation, only *GhTLP12A.1 cis*-regulatory elements comprised an MBSI *cis*-regulatory element related to flavonoid biosynthetic regulatory genes, which are very crucial to provide drought tolerance in wheat (Ma D. Y. et al., 2014). Overexpressing many of the genes of flavonoid pathways also provides tolerance under salt stress (Ashraf, 2009; Yang et al., 2009; Matus et al., 2010; Le Martret et al., 2011; Bharti et al., 2015). *GhTLP11A* comprised the higher number of salicylic acid (SA)-responsive TCA elements. Salicylic acid was identified as a potential hormone to provide tolerance against salinity (Khan et al., 2012) and improves the drought tolerance in rice (Farooq et al., 2009).

*GhTLP5A.2, GhTLP5D.2, GhTLP11A*, and *GhTLP12A.1* also showed significant relative expression in qRT-PCR. From these observations, it could be speculated that the proximal elements of *GhTLP11A* and *GhTLP12A.1* might have an important role in controlling the regulation and improvement of salt- and drought-stress responses in cotton. The results of the metabolic pathway study of PCoEGs and NCoEGs of *GhTLP11A* and *GhTLP12A.1* genes and *cis*-regulatory elements also provided evidence of the involvement of two of these genes in salt- and drought-stress responses. However, detailed molecular explorations are required to understand the structural-functional relationship of cotton *TLP* genes and the involvement of *GhTLPs* to enhance the tolerance against drought and salt stresses.

## Conclusion

In this study, a total of 105 cotton TLP proteins with a highly conserved tubby domain at C-terminal and N-terminal F-box were identified in four cotton species (*G. arboreum, G. raimondii, G. hirsutum*, and *G. barbadense*). Their protein domains, conserved motifs, and gene structure within the same groups shared a notable similarity, which leads to some similar functions. Furthermore, the cotton *TLP12* gene family members clustered into 4 and 8 groups and experienced higher evolutionary pressure in comparison to others, showing their functional divergence in *Gossypium* species. Several *G. hirsutum TLP* genes showed significantly high expression in both drought- and salt-stress conditions. Two genes *GhTLP11A* and *GhTLP12A.1* demonstrated comparatively higher expression and provided strong evidence that these genes can play a predominant role during drought and salt stress. Our investigation enhances the understanding of *TLP* genes in cotton at the level of function, evolution, and structure, which further highlights the intriguing field of *TLP* genes that have immense prospects for future manipulation.

## Data Availability Statement

The datasets presented in this study can be found in online repositories. The names of the repository/repositories and accession number(s) can be found at: NCBI (https://www.ncbi.nlm.nih.gov/geo/) under accession numbers PRJNA532694.

## Author Contributions

NB carried out the bioinformatics analysis and design and drafted the manuscript. SF performed quantitative expression analysis. CM and SB participated to supervise the study. All the authors have read and approved the final manuscript.

## Conflict of Interest

The authors declare that the research was conducted in the absence of any commercial or financial relationships that could be construed as a potential conflict of interest.

## Supplementary Material

Supplementary Figure 1 Diagrammatic representation of the functional domain of GaTLPs, GrTLPs, GhTLPs, and GbTLPs. Bioinformatics study of conserved domains was done by the NCBI Batch CD-search tool.

Supplementary Figure 2 Multiple sequence alignment of all identified *TLP* genes in cotton (*Gossypium arboreum, Gossypium raimondii, Gossypium hirsutum*, and *Gossypium barbadense)*. The conserved residues are shown by dark background.

Supplementary Figure 3 Cotton *TLP12* genes phylogenetic tree with different eudicots. Cotton *TLP12* genes (*GaTLPs12, GrTLPs12, GhTLPs12A, GhTLPs12D, GbTLPs12A*, and *GbTLPs12D*) with different eudicots were aligned with each other. The phylogenetic tree ML was built, using 1,000 bootstrap values.

Supplementary Figure 4 Co-expression network analysis of *GhTLP11A* and *GhTLP12A.1* genes. Positively and negatively co-expressed genes (PCoEGs and NCoEGs) with *GhTLP11A* and *GhTLP12A.1* in salt and drought stress at different time scales. Rhombus (nodes) represents transcripts, and lines (edges) represent transcriptional interaction between *GhTLPs* and transcripts.

Supplementary Figure 5 PageMan-based functional classification of Positively and negatively co-expressed genes (PCoEGs and NCoEGs) in transcriptional regulation, signaling, lipid metabolism, stress, signaling, secondary and hormone metabolism in **(A,B)** salt- and **(C,D)** drought-stress condition at 0, 6, 12, 24, 48, and 72 h. BINs colored in green and red are positively and negatively regulated groups, respectively. Expression values in the log 2 scale indicated by the scale bar.

Supplementary Table 1 Reciprocal blast of cotton and *A. thaliana* TLP proteins.

Supplementary Table 2 A list of eudicots plants used for groups 4 and 8 cotton *TLP* genes (*TLP12*) phylogenetic analysis.

Supplementary Table 3 Fifteen conserved protein motif sequences and their annotation in cotton *TLPs*.

Supplementary Table 4 Orthologous *TLP* gene pairs among *Gossypium* taxa with their chromosomal location and percentage identity.

Supplementary Table 5 The molecular evolutionary rates (*Ka, Ks, Ka/Ks* ratios) of orthologous *TLP* gene pairs among *Gossypium* taxa.

Supplementary Table 6 A list of positively and negatively co-expressed genes (Pearson correlation coefficient *r* ≥ 0.95/*r* ≤ −0.95) with *GhTLP711A* and *GhTLP12A.1* in salt stress.

Supplementary Table 7 A list of positively and negatively co-expressed genes (Pearson correlation coefficient *r* ≥ 0.95/*r* ≤ −0.95) with *GhTLP711A* and *GhTLP12A.1* in drought stress.

Supplementary Table 8 Identified miRNAs and their targets.

Supplementary Table 9 Distribution of *cis*-regulatory elements in the promoter regions of *GhTLP* genes and their annotations.
