# Supplementary figures and images for: Genome-Wide Identification and Evolutionary Analysis of Gossypium Tubby-Like Protein (TLP) Gene Family and Expression Analyses During Salt and Drought Stress
Source: Front Plant Sci. 2021 Jul 21;12:667929. doi: 10.3389/fpls.2021.667929 (PMC8335595; doi:10.3389/fpls.2021.667929)

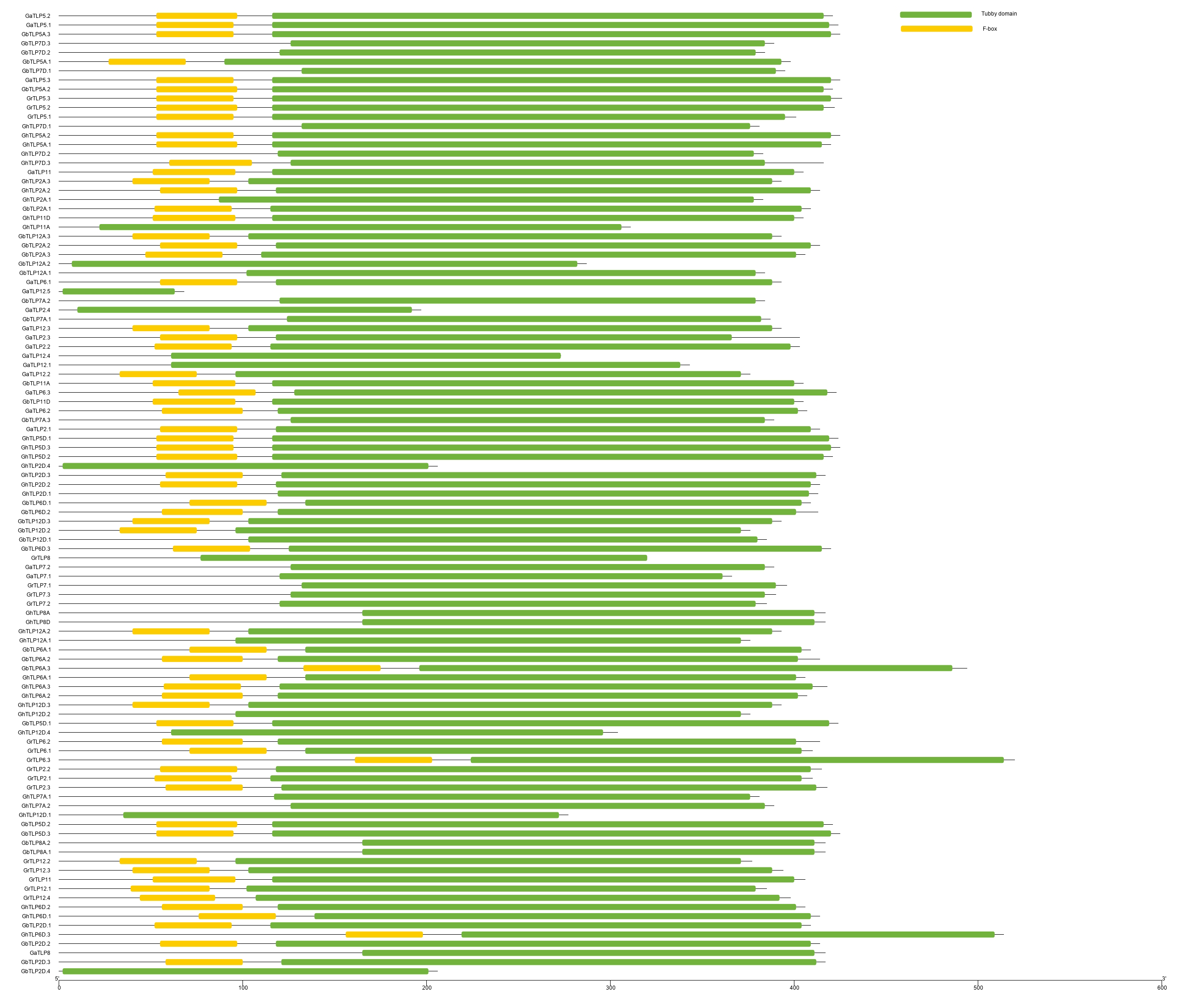

Supplement: Supplementary Figure 1 — Diagrammatic representation of the functional domain of GaTLPs, GrTLPs, GhTLPs, and GbTLPs. Bioinformatics study of conserved domains was done by the NCBI Batch CD-search tool. [file Image_1.tif]

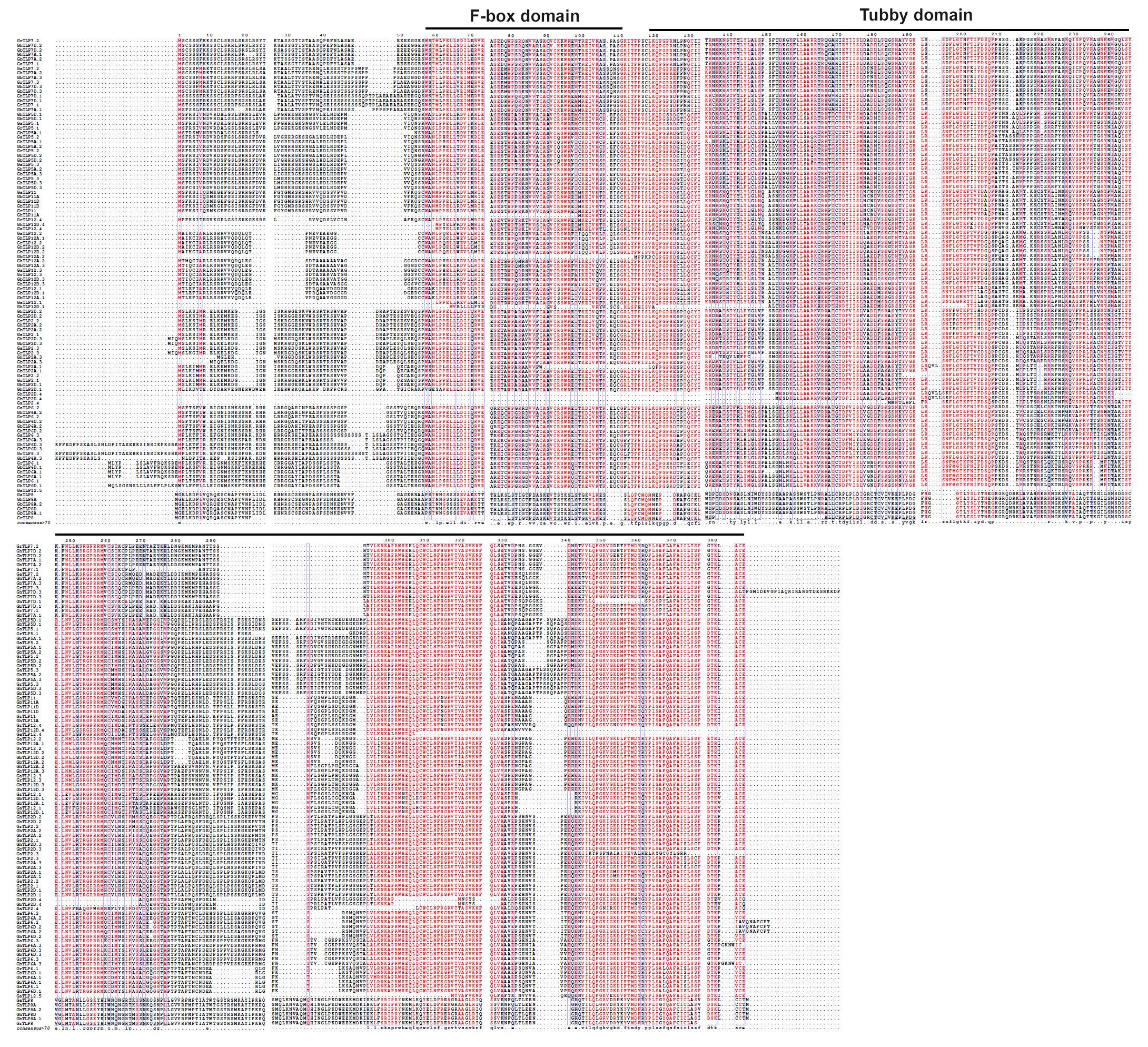

Supplement: Supplementary Figure 2 — Multiple sequence alignment of all identified TLP genes in cotton (Gossypium arboreum, Gossypium raimondii, Gossypium hirsutum, and Gossypium barbadense). The conserved residues are shown by dark background. [file Image_2.tif]

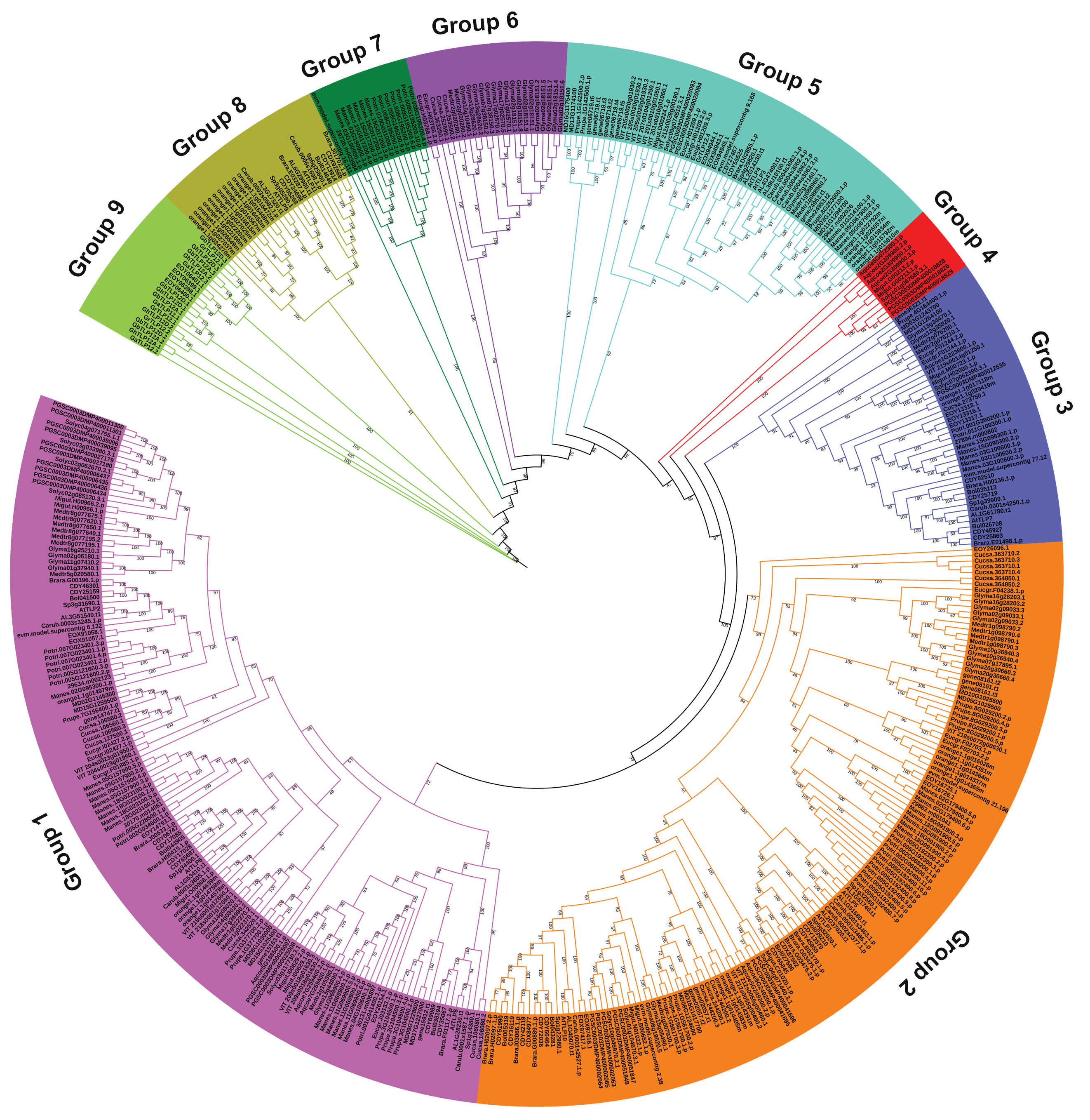

Supplement: Supplementary Figure 3 — Cotton TLP12 genes phylogenetic tree with different eudicots. Cotton TLP12 genes (GaTLPs12, GrTLPs12, GhTLPs12A, GhTLPs12D, GbTLPs12A, and GbTLPs12D) with different eudicots were aligned with each other. The phylogenetic tree ML was built, using 1,000 bootstrap values. [file Image_3.tif]

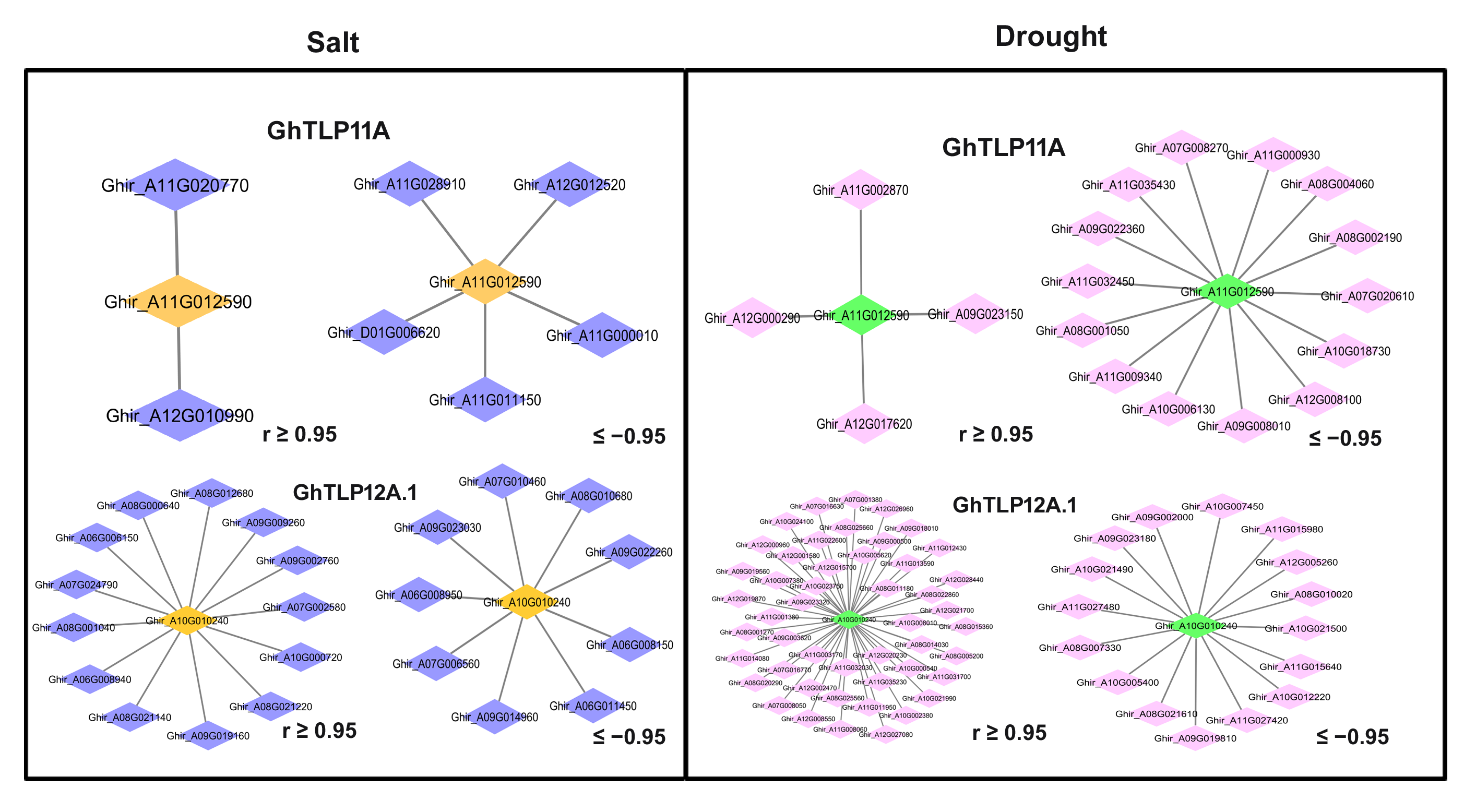

Supplement: Supplementary Figure 4 — Co-expression network analysis of GhTLP11A and GhTLP12A.1 genes. Positively and negatively co-expressed genes (PCoEGs and NCoEGs) with GhTLP11A and GhTLP12A.1 in salt and drought stress at different time scales. Rhombus (nodes) represents transcripts, and lines (edges) represent transcriptional interaction between GhTLPs and transcripts. [file Image_4.tif]

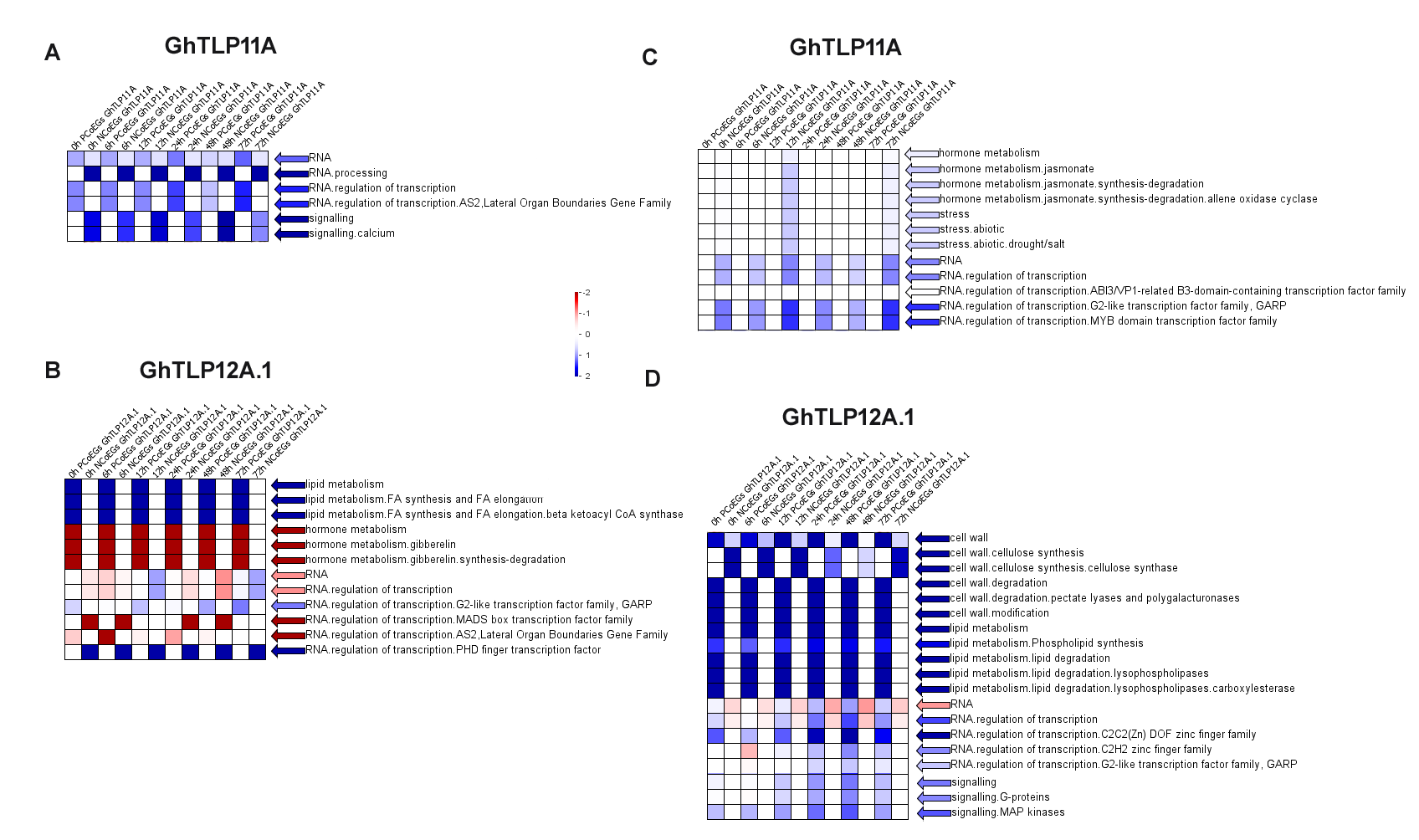

Supplement: Supplementary Figure 5 — PageMan-based functional classification of Positively and negatively co-expressed genes (PCoEGs and NCoEGs) in transcriptional regulation, signaling, lipid metabolism, stress, signaling, secondary and hormone metabolism in (A,B) salt- and (C,D) drought-stress condition at 0, 6, 12, 24, 48, and 72 h. BINs colored in green and red are positively and negatively regulated groups, respectively. Expression values in the log 2 scale indicated by the scale bar. [file Image_5.tif]
